# Safety and pharmacokinetics of single, dual, and triple antiretroviral drug formulations delivered by pod-intravaginal rings designed for HIV-1 prevention: A Phase I trial

**DOI:** 10.1371/journal.pmed.1002655

**Published:** 2018-09-28

**Authors:** Kathleen L. Vincent, John A. Moss, Mark A. Marzinke, Craig W. Hendrix, Peter A. Anton, Richard B. Pyles, Kate M. Guthrie, Lauren Dawson, Trevelyn J. Olive, Irina Butkyavichene, Scott A. Churchman, John M. Cortez, Rob Fanter, Manjula Gunawardana, Christine S. Miller, Flora Yang, Rochelle K. Rosen, Sara E. Vargas, Marc M. Baum

**Affiliations:** 1 Department of Obstetrics and Gynecology, University of Texas Medical Branch, Galveston, Texas, United States of America; 2 Department of Chemistry, Oak Crest Institute of Science, Monrovia, California, United States of America; 3 Division of Clinical Pharmacology, Department of Medicine, Johns Hopkins University, Baltimore, Maryland, United States of America; 4 Department of Pathology, Johns Hopkins University, Baltimore, Maryland, United States of America; 5 Center for HIV Prevention Research, Division of Digestive Diseases and UCLA AIDS Institute, David Geffen School of Medicine, University of California Los Angeles, Los Angeles, California, United States of America; 6 Department of Pediatrics, University of Texas Medical Branch, Galveston, Texas, United States of America; 7 Department of Microbiology and Immunology, University of Texas Medical Branch, Galveston, Texas, United States of America; 8 The Centers for Behavioral & Preventive Medicine, The Miriam Hospital, Providence, Rhode Island, United States of America; 9 Department of Psychiatry & Human Behavior, Alpert Medical School of Brown University, Providence, Rhode Island, United States of America; 10 Department of Behavioral & Social Sciences, Brown University School of Public Health, Providence, Rhode Island, United States of America; Desmond Tutu HIV Centre, SOUTH AFRICA

## Abstract

**Background:**

Intravaginal rings (IVRs) for HIV pre-exposure prophylaxis (PrEP) theoretically overcome some adherence concerns associated with frequent dosing that can occur with oral or vaginal film/gel regimens. An innovative pod-IVR, composed of an elastomer scaffold that can hold up to 10 polymer-coated drug cores (or “pods”), is distinct from other IVR designs as drug release from each pod can be controlled independently. A pod-IVR has been developed for the delivery of tenofovir (TFV) disoproxil fumarate (TDF) in combination with emtricitabine (FTC), as daily oral TDF-FTC is the only Food and Drug Administration (FDA)-approved regimen for HIV PrEP. A triple combination IVR building on this platform and delivering TDF-FTC along with the antiretroviral (ARV) agent maraviroc (MVC) also is under development.

**Methodology and findings:**

This pilot Phase I trial conducted between June 23, 2015, and July 15, 2016, evaluated the safety, pharmacokinetics (PKs), and acceptability of pod-IVRs delivering 3 different ARV regimens: 1) TDF only, 2) TDF-FTC, and 3) TDF-FTC-MVC over 7 d. The crossover, open-label portion of the trial (*N* = 6) consisted of 7 d of continuous TDF pod-IVR use, a wash-out phase, and 7 d of continuous TDF-FTC pod-IVR use. After a 3-mo pause to evaluate safety and PK of the TDF and TDF-FTC pod-IVRs, TDF-FTC-MVC pod-IVRs (*N* = 6) were evaluated over 7 d of continuous use. Safety was assessed by adverse events (AEs), colposcopy, and culture-independent analysis of the vaginal microbiome (VMB). Drug and drug metabolite concentrations in plasma, cervicovaginal fluids (CVFs), cervicovaginal lavages (CVLs), and vaginal tissue (VT) biopsies were determined via liquid chromatographic-tandem mass spectrometry (LC-MS/MS). Perceptibility and acceptability were assessed by surveys and interviews. Median participant age was as follows: TDF/TDF-FTC group, 26 y (range 24–35 y), 2 White, 2 Hispanic, and 2 African American; TDF-FTC-MVC group, 24.5 y (range 21–41 y), 3 White, 1 Hispanic, and 2 African American. Reported acceptability was high for all 3 products, and pod-IVR use was confirmed by residual drug levels in used IVRs. There were no serious adverse events (SAEs) during the study. There were 26 AEs reported during TDF/TDF-FTC IVR use (itching, discharge, discomfort), with no differences between TDF alone or in combination with FTC observed. In the TDF-FTC-MVC IVR group, there were 12 AEs (itching, discharge, discomfort) during IVR use regardless of attribution to study product. No epithelial disruption/thinning was seen by colposcopy, and no systematic VMB shifts were observed. Median (IQR) tenofovir diphosphate (TFV-DP) tissue concentrations of 303 (277–938) fmol/10^6^ cells (TDF), 289 (110–603) fmol/10^6^ cells (TDF-FTC), and 302 (177.1–823.8) fmol/10^6^ cells (TDF-FTC-MVC) were sustained for 7 d, exceeding theoretical target concentrations for vaginal HIV prevention. The study’s main limitations include the small sample size, short duration (7 d versus 28 d), and the lack of FTC triphosphate measurements in VT biopsies.

**Conclusions:**

An innovative pod-IVR delivery device with 3 different formulations delivering different regimens of ARV drugs vaginally appeared to be safe and acceptable and provided drug concentrations in CVFs and tissues exceeding concentrations achieved by highly protective oral dosing, suggesting that efficacy for vaginal HIV PrEP is achievable. These results show that an alternate, more adherence-independent, longer-acting prevention device based on the only FDA-approved PrEP combination regimen can be advanced to safety and efficacy testing.

**Trial registration:**

ClinicalTrials.gov NCT02431273

## Abstract 2

In a Phase I trial, Kathleen Vincent and colleagues study the safety and acceptability of a pod-intravaginal ring able to supply multiple antiretroviral drugs.

## Author summary

**Why was this study done?:**

- Pre-exposure Prophylaxis (PrEP) for HIV prevention has been shown to be effective in highly adherent users.
- Preclinical studies of an innovative pod-intravaginal ring (IVR) design delivering antiretroviral (ARV) regimens have shown protection against Simian Human Immunodeficiency Virus (SHIV) in nonhuman primates.
- The goal of this clinical study was to assess safety, pharmacokinetics (PKs), and acceptability of pod-IVRs delivering 3 different ARV regimens: 1) tenofovir disoproxil fumarate (TDF) only, 2) TDF and emtricitabine (FTC), and 3) TDF, FTC, and maraviroc (MVC) via the vaginal route.

**What did the researchers do and find?:**

- A 7-d vaginal ring study was performed, sequentially delivering TDF, TDF-FTC, and TDF-FTC-MVC in 6 women to determine safety and PKs.
- There were no concerning safety findings that would inhibit further development of the vaginal rings.
- Vaginal secretion and tissue levels were detected in ranges that have been associated with protection against HIV/SHIV infection in preclinical and clinical studies.

**What do these findings mean?:**

- Vaginal delivery of combinations of ARV drugs, including the only Centers for Disease Control (CDC)-recommended regimen for HIV PrEP, was achieved in this early Phase I clinical study.
- The interventions appeared safe and acceptable and therefore merit further study.

## Introduction

Scale-up of prevention and treatment efforts to curb the HIV epidemic have resulted in decreasing the number of new infections by half per year in 2012 since the peak in 1996 [1]. In Fast-Track, the Joint United Nations Programme on HIV/AIDS (UNAIDS) has set aggressive goals, including 500,000 (or fewer) new annual infections by 2020, a 75% reduction from 2010 numbers, and an end to the AIDS epidemic by 2030 [2]. Unfortunately, the number of annual, new HIV infections has stalled around 1.9 million since 2010, suggesting that a prevention gap has been reached [3]. To meet these ambitious UNAIDS Fast-Track goals, further work is needed to develop new highly effective strategies for HIV prevention.

Pre-exposure prophylaxis (PrEP) using Food and Drug Administration (FDA)-approved antiretroviral (ARV) drugs holds significant promise as a strategy for preventing HIV infection. Multiple HIV PrEP clinical trials have demonstrated that vaginal and oral ARV regimens based on the nucleoside reverse transcriptase inhibitor (NRTI) tenofovir (TFV), alone or in combination with the NRTI emtricitabine (FTC), can be effective in susceptible men, women, and partners of HIV-infected individuals [4–11]. Oral tenofovir disoproxil fumarate (TDF)-FTC (Truvada, Gilead Sciences, Foster City, CA) is the only FDA-approved regimen for HIV PrEP [12]. Centre for the AIDS Programme of Research in South Africa (CAPRISA)-004 demonstrated that topical dosing—in this case pericoitally with a 1% TFV gel—can be effective in preventing vaginal HIV transmission [4]. In post hoc analyses of both Vaginal and Oral Interventions to Control the Epidemic (VOICE) gel and Follow-on African Consortium for Tenofovir Studies (FACTS) 001 trials, TFV gel was effective in highly adherent women [13]. A critical factor driving success in these trials appears to involve sustaining high adherence to frequent dosing [14].

Treatment of any medical condition that requires long-term use of medications must address the challenge to maintain adherence to therapy; adherence has been shown to be inversely related to dosing frequency regardless of dosing formulation [15–18]. Use of extended-release or long-acting formulations, including intravaginal rings (IVRs) [19], implantable devices, and injectable formulations [20], can decrease the burden of frequent dosing and potentially improve adherence. Two Phase 3, randomized, double-blind, placebo-controlled trials (MTN-020–A Study to Prevent Infection with a Ring for Extended Use (ASPIRE) and IPM 027–The Ring Study) recently evaluated a monthly IVR delivering the non-nucleoside HIV reverse-transcriptase inhibitor dapivirine (DPV) [21,22]. The trials enrolled 2,629 and 1,959 women, respectively, between the ages of 18 and 45 years in Malawi, South Africa, Uganda, and Zimbabwe and demonstrated that an IVR delivering an ARV agent can reduce the risk of HIV acquisition by as much as 56% in highly adherent users, as determined through quarterly plasma and residual IVR DPV concentrations.

User adherence to IVRs depends on a number of factors including user sensory perceptions and experiences of product characteristics and impact such as dimensionality and perceptions of increased discharge [23]. Users ascribe meaning to product characteristics, and those meanings can influence perceptions of product efficacy and the user’s willingness to engage in consistent and correct product use [23,24].

The primary purpose of this exploratory, open label clinical trial was to evaluate the safety, pharmacokinetics (PKs), and user perceptibility of an innovative IVR design (termed “pod-IVR”) [25–27] delivering three formulations: first, TDF alone over 7 d; second, TDF-FTC over 7 d in a crossover design; and third, TDF-FTC-maraviroc (MVC) over 7 d. The pod-IVR consists of a silicone scaffold that holds up to 10 individual “pods” of polymer-coated drug cores, allowing independently controlled drug release from each pod through delivery channels.

## Materials and methods

### Ethics statement

All human research was approved by the University of Texas Medical Branch Institutional Review Board (IRB #14–0479), conducted according to the Declaration of Helsinki, and registered in clinicaltrials.gov (NCT02431273). All participants provided written informed consent.

### Clinical trial design

Women were recruited through announcements to use IVRs releasing TDF, TDF-FTC, and TDF-FTC-MVC for 7 d each between June 23, 2015, and July 15, 2016. Women were prescreened to confirm basic eligibility and then scheduled for a screening visit where inclusion/exclusion criteria were reviewed, medical and sexual history were obtained, and baseline labs with blood counts, liver and kidney function, and sexually transmitted infection (STI) and HIV screening were collected. Inclusion criteria were women aged 18–45 with regular or suppressed menses, on contraception, and willing to abstain from sexual intercourse during IVR use. Women with STIs, HIV, Hepatitis B, abnormal screening labs, allergy to study product, currently using an IVR, or at high risk for HIV were excluded.

The trial consisted of an open-label, crossover design of a pod-IVR with 3 different ARV regimens. There were 3 separate 7-d treatment periods (Treatment Period 1, TDF-only pod-IVR; Treatment Period 2, TDF-FTC pod-IVR; and Treatment Period 3, TDF-FTC-MVC pod-IVR) with 6 participants per treatment period. Each participant used each pod-IVR for 7 d with a washout period of at least 14 d between each treatment period. Progression to the next treatment period was contingent upon the absence of grade 3 or 4 genitourinary adverse events (AEs) considered to be drug related by the investigator or other investigator-assessed drug-related serious adverse event (SAE). There was a 3-mo pause and re-consent between the second and third treatment periods to allow for determination of safety and PKs of the combination IVR before proceeding to the use of the TDF-FTC-MVC pod-IVR in the third treatment period. The study design was chosen with the ultimate goal of developing a TDF-FTC and TDF-FTC-MVC pod-IVR; however, the FDA required assessment of a TDF-only pod-IVR prior to use of the TDF-FTC and TDF-FTC-MVC formulations.

Following the screening visit, women who were clinically deemed eligible returned to the clinic for IVR insertion (Visit 1, TDF IVR; Visit 5, TDF-FTC IVR) after cessation of menses. Women were instructed on vaginal ring placement, which was performed during this visit, and were asked to be abstinent during IVR use. They returned for Visit 2 (TDF IVR) or Visit 6 (TDF-FTC IVR) on Day 2 (± 1 d) after IVR insertion and for Visit 3 (TDF IVR) or Visit 7 (TDF-FTC IVR) on Day 7 (± 1 d) when the IVRs were removed. Women returned for follow-up 1–2 wk after IVR removal for Visit 4 (TDF IVR) or Visit 8 (TDF-FTC IVR).

The TDF-FTC-MVC pod-IVR arm was carried out separately from the TDF and TDF-FTC IVR portion of the study. Women who were deemed clinically eligible returned to the clinic for IVR insertion (Visit 1) after cessation of menses, instructed on vaginal ring placement (which was performed during this visit), and asked to be abstinent during IVR use. Women returned for Visit 2 on Day 2 (± 1 d) after IVR insertion and for Visit 3 on Day 7 (± 1 d) when the IVRs were removed. Women returned for follow-up 1–2 wk after IVR removal for Visit 4.

During all visits, AEs were reviewed and colposcopy was performed to evaluate the vagina and cervix for safety assessments. For PK assessments, blood (plasma) and cervicovaginal samples (cervicovaginal fluid [CVF], Dacron swabs; cervicovaginal lavage [CVL], 2.5 mL sterile phosphate-buffered saline solution containing 1 mM LiCl) were collected. In summary, for PK assessments, two samples were collected during pod-IVR use (Visit 2/6/2 and Visit 3/7/3) and one sample after pod-IVR removal (Visit 4/8/4). Additionally, at Visit 3/7/3, the IVRs were collected and vaginal biopsies were obtained. One biopsy was flash-frozen in liquid nitrogen for the analysis of ARV drug concentrations. A second vaginal biopsy was fixed in formalin and hematoxylin–eosin stained. The slides were reviewed by a trained pathologist with expertise in reproductive tract pathology.

### User perception and product acceptability

Behavioral assessments of perceptibility, acceptability, and adherence were obtained through daily diaries during IVR use, surveys completed using computer-assisted self-interview (CASI) format at the time of IVR removal, and qualitative interviews conducted at Visit 4/Visit 8/Visit 4. Baseline demographics and sexual history surveys were completed prior to product initiation. A user sensory perception and experience (USPE) survey, acceptability and adherence measures, and an in-depth interview were completed following IVR use.

### Chemicals and reagents

TDF and FTC labeled for human use were purchased from commercial vendors with a Drug Master File (DMF) registered with the FDA. MVC was isolated from the commercial formulation (Pfizer, Inc., New York, NY), which consists of film-coated tablets for oral administration containing 300 mg of MVC and inactive ingredients, as described previously [26]. All other reagents were obtained from Sigma-Aldrich, unless otherwise noted.

### Fabrication of pod-intravaginal rings

Polydimethylsiloxane ([PDMS], silicone) pod-IVRs were fabricated in a multistep process that has been described in detail elsewhere [25,26,28,29], and only a brief description is provided here. Each pod contained a single drug. The drug powder was compacted into cores of 3.2-mm outer diameter in a manual tablet press (MTCM-I; Globe Pharma, New Brunswick, NJ). Drug cores were coated with poly(vinyl alcohol) to yield pods, which were placed in the corresponding IVR cavities and sealed in place by back-filling with room-temperature-cure silicone. Each pod was matched with the appropriate configuration of mechanically punched delivery channels. The IVR drug loadings were as follows: TDF, 180 mg; FTC, 125 mg; MVC, 90 mg. Residual drug content of used IVRs was measured by high-performance liquid chromatography (HPLC) according to published methods [26,28] and used to calculate in vivo release rates.

### Safety measures

AEs were reviewed at all study visits along with colposcopy. Histology was performed on VT samples collected on Day 7 (Visit 3/7/3). Vaginal pH and Nugent scores [30] were measured at each study visit. Vaginal microbial community profiles also were measured at each study visit by a custom quantitative polymerase chain reaction (qPCR) array described previously [31]. The array targets 46 distinct key vaginal microbiota to the species level along with several housekeeping genes.

### Bioanalysis of in vivo samples

Concentrations of TDF, TFV, tenofovir diphosphate (TFV-DP), FTC, and MVC were determined via previously described liquid chromatographic-tandem mass spectrometric (LC-MS/MS) assays [32–35]. All assays were developed and validated following the FDA Guidance for Industry, Bioanalytical Method Validation recommendations and met all acceptability criteria [36]. Isotopically labeled internal standards were used for all compounds and the determination of drug concentrations in all specimen sources.

The lower limits of quantification for these methods were as follows: CVFs, TDF (0.0625 ng/sample), TFV (0.25 ng/sample), FTC (1.0 ng/sample), MVC (0.05 ng/sample); CVL, TDF (0.5 ng/mL), TFV (5.0 ng/mL), FTC (20 ng/ mL), MVC (1 ng/mL); VT homogenate, TFV (0.05 ng/sample), TFV-DP (50 fmol/sample), FTC (0.25 ng/sample), MVC (0.05 ng/sample); plasma, TFV (0.31 ng/mL), FTC (0.31 ng/mL), MVC (0.1 ng/mL). CVF and tissue samples were ultimately reported as ng/mg or fmol/mg, respectively, following normalization to net biopsy or CVF weight. VT TDF concentrations were not measured.

CVLs were performed by instilling a sterile phosphate-buffered saline solution (2.5 mL) containing LiCl (1 mM). The added LiCl allowed dilution of the collected CVF to be calculated in the TDF-FTC-MVC group using an established method [37]. CVL samples were not compensated for dilution in the TDF and TDF-FTC groups.

### PK analysis

PK parameter values for CVF were determined by noncompartmental analysis (NCA) using Phoenix WinNonlin 6.4 (Pharsight Corporation, Sunnyvale, CA). The NCA was run using the linear trapezoidal rule for increasing concentration data and the logarithmic trapezoidal rule for decreasing concentration data (linear up and log down) as the calculation method. Post-dose concentrations below the corresponding lower limit of quantification (LLOQ) (*C*_*LLQ*_) were imputed as follows:

$$C_{LLQ}=\frac{LLOQ\;of\;assay}{2\times (median\;sample\;mass)}$$ (1)

### Data analysis

Qualitative data were analyzed by applying study-specific thematic identification and summarizing verbatim transcript data per theme, with illustrative quotes retained as conventional [38]. Quantitative data were analyzed using GraphPad Prism (version 7.00, GraphPad Software, Inc., La Jolla, CA). Statistical significance is defined as two-sided *P* < 0.05. The unpaired, nonparametric Mann–Whitney test was used to compare two groups, including vaginal pH, Nugent scores, in vivo TDF release rates, CVF FTC concentrations in the TDF-FTC and TDF-FTC-MVC pod-IVR treatment periods and terminal half-lives of ARV drug elimination from CVF within one IVR treatment period and across IVR treatment periods. The nonparametric (i.e., do not assume a Gaussian distribution) Kruskal–Wallis tests with no matching/pairing of the data was used to compare three IVRs, including CVF TDF and TFV concentrations and CVF:VT concentration ratios and terminal half-lives of ARV drug elimination from CVF within one IVR arm.

## Results

### Study participants

Six participants were enrolled and completed the first 2 treatment periods using the TDF-only and TDF-FTC pod-IVRs. Due to the delay between treatment periods 2 and 3, 4 participants dropped out and were replaced for treatment period 3. Demographics for the initial 6 study participants who completed the first 2 treatment periods (i.e., TDF-only pod-IVR and TDF-FTC pod-IVR) can be found in Table 1, and demographics for the final treatment period (i.e., TDF-FTC-MVC pod-IVR) can be found in Table 2. There were no missed study visits, and one participant (ID 479–12) had an additional visit (Visit 2A, TDF pod-IVR arm) with visits on Day 1 (Visit 2) and Day 2 (Visit 2A) for evaluation of AEs (see Safety measures below).

**Table 1:** Demographics of study participants completing the first 2 treatment periods.

Participants enrolled, *n* (percent)	6 (100)
Age (years), median (range)	26 (24–35)
Race and Ethnicity, *n* (percent)	
Black or African American	2 (33)
White	4 (67)
Hispanic	2 (33)
Non-Hispanic	2 (33)
Asian	0 (0)
Other	0 (0)
Tobacco use, *n* (percent)	1 (17)
Alcohol use, *n* (percent)	4 (67)
BMI (kg/m^2^), median (range)	32.4 (27.2–44.2)

**Abbreviation:** BMI, body mass index.

**Table 2:** Demographics of study participants completing the 3rd treatment period.

Participants enrolled, *n* (percent)	6 (100)
Age (years), median (range)	24.5 (21–41)
Race and Ethnicity, *n* (percent)	
Black or African American	2 (33)
White	4 (67)
Hispanic	1 (17)
Non-Hispanic	3 (50)
Asian	0 (0)
Other	0 (0)
Tobacco use, *n* (percent)	1 (17)
Alcohol use, *n* (percent)	5 (83)
BMI (kg/m^2^), median (range)	28.4 (23.8–44.6)

**Abbreviation:** BMI, body mass index.

### Safety measures

The pod-IVRs were safe and generally well tolerated in all three treatment periods. AEs were recorded with daily diaries and during study visits. There were no concerning safety findings by participant report, examination with colposcopy, evaluation of vaginal microbiome (VMB), or histology from vaginal biopsy. There were no SAEs or grade 3 or 4 genitourinary AEs. Fifty-eight AEs occurred during the study period (54 Grade 1, 4 Grade 2; Tables 3–5). All AEs were Grade 1 except for four Grade 2 findings. Two of the Grade 2 events were pelvic pain/cramping, for which the subjects took over-the counter medication and were determined to be "possibly related." One woman had a Grade 2 candida vaginitis determined to be "possibly related," which did not recur after treatment. One woman reported having Grade 2 diarrhea, which was determined to be "not related" and attributed to “food poisoning”.

**Table 3:** AEs during TDF pod-IVR use.

AE	Number of Participants experiencing AEs	Number of AEs reported	Grade of AE	Relatedness to IVR use
Pelvic Pain	3	5	Grade 1 (*n* = 4)* Grade 2 (*n* = 1)**	Possibly Related
Vaginal Discharge	3	3	Grade 1 (all)	Possibly Related
Metrorrhagia	1	1	Grade 1	Possibly Related
Vulvovaginal Itching	3	3	Grade 1 (all)	Possibly Related
Cervicovaginal Erythema	3	3	Grade 1 (all)	Possibly Related
Diarrhea	1	1	Grade 1	Possibly Related
Malaise	1	1	Grade 1	Not Related

*Grade 1 AEs are mild and cause no or minimal interference with activity.

**Grade 2 AEs are controlled with medication or cause greater than minimal interference with activity. The AE was considered Grade 2 because the subject used over-the-counter medication for treatment.

**Abbreviations:** AE, adverse event; IVR, intravaginal ring; TDF, tenofovir disoproxil fumarate.

**Table 4:** AEs during TDF-FTC pod-IVR use.

AE	Number of Participants experiencing AEs	Number of AEs reported	Grade of AE	Relatedness to IVR use
Pelvic Pain	2	3	Grade 1 (*n* = 1)* Grade 2 (*n* = 1)**	Possibly Related
Vaginal Discharge	3	3	Grade 1 (all)	Possibly Related
Cervicovaginal Erythema	1	3	Grade 1 (all)	Possibly Related

*Grade 1 AEs are mild and cause no or minimal interference with activity.

**Grade 2 AEs are controlled with medication or cause greater than minimal interference with activity. The AE was considered Grade 2 because the subject used over-the-counter medication for treatment.

**Abbreviations:** AE, adverse event; FTC, emtricitabine; IVR, intravaginal ring; TDF, tenofovir disoproxil fumarate.

**Table 5:** AEs during TDF-FTC-MVC pod-IVR use.

AE	Number of Participants experiencing AEs	Number of AEs reported	Grade of AE	Relatedness to IVR use
Pelvic Pain	4	5	Grade 1*	Possibly Related
Vaginal Discharge	1	1	Grade 1	Possibly Related
Metrorrhagia	2	2	Grade 1	Possibly Related
Vulvovaginal Itching	1	1	Grade 1	Possibly Related
Odor	1	1	Grade 1	Possibly Related
Nausea	1	1	Grade 1	Possibly Related
*Candida* (monilial vulvovaginitis)	1	1	Grade 2**	Possibly Related

*Grade 1 AEs are mild and cause no or minimal interference with activity.

**Grade 2 AEs are controlled with medication or cause greater than minimal interference with activity. The AE was considered Grade 2 because the subject had moderate symptoms.

**Abbreviations:** AE, adverse event; FTC, emtricitabine; IVR, intravaginal ring; MVC, maraviroc; TDF, tenofovir disoproxil fumarate.

The following is a description of findings during IVR use. Thirty-eight AEs occurred during use of the IVRs, 17 during TDF IVR use (Table 3), 9 during TDF-FTC use (Table 4), and 12 during TDF-FTC-MVC use (Table 5). On colposcopy, cervicovaginal erythema was found in 3 women (6 findings) during IVR use, 2 of whom had erythema at baseline. During IVR use, 6 women had pelvic pain/cramping (13 findings). Four women reported vaginal discharge (7 findings); one episode was a mucus-like discharge that appeared peri-ovulatory that was deemed "possibly related." Three women had metrorrhagia/intermenstrual bleeding during IVR use (3 findings), one during TDF IVR use and two during TDF-FTC-MVC use. Three women had vulvovaginal itching (3 findings). Two women were diagnosed with candida vaginitis (one Grade 1 and one Grade 2) and were treated. One woman reported an odor (1 finding) on Day 7 of TDF-FTC-MVC IVR use. One woman reported nausea (1 finding) associated with cramping which lasted only 10 min during TDF-FTC-MVC IVR use. One woman had Grade 1 diarrhea during TDF-only IVR use, which was "possibly related." One woman had malaise after influenza vaccine during TDF-only IVR use, and this was considered "not related."

With two exceptions, all biopsies were reported as “consistent with a normal vaginal epithelium”. For one subject in the TDF pod-IVR treatment period, there was one section of the vaginal biopsy with minimal to mild inflammatory infiltrates and necrosis, which may be consistent with an infection (she had asymptomatic *Candida* infection determined by qPCR); a recut from the same specimen showed normal tissue. In the TDF-FTC-MVC pod-IVR treatment period, there was a finding of minimal inflammatory infiltrate and hemorrhage in the subject who had the Grade 2 symptomatic *Candida* infection (Table 5).

In all 3 treatment periods, the impact of the pod-IVRs on the vaginal microbial community profiles was analyzed using custom qPCR arrays targeting the most common 46 vaginal bacteria at genus or species levels [31]. A second array was used to quantify the levels of 16 additional pathogen targets, including bacteria and viruses common to the vagina. Collectively, the results show no clear, systematic impact of the pod-IVRs on the stability of the microbial community profiles for the trial participants (S1–S3 Figs). One subject (ID 479–16) had asymptomatic *Candida albicans* noted only by qPCR (saline microscopy with potassium hydroxide [KOH] was negative) during the study.

The median vaginal pH prior to IVR placement (Visits 0 and 1) was 4.0 (IQR, 4.0–4.5), and with the TDF and TDF-FTC pod-IVRs in place was 4.5 (IQR, 4.1–4.5) and 4.0 (IQR, 4.0–4.5), respectively. There was no statistically significant difference in vaginal pH prior to pod-IVR placement compared to during pod-IVR use: TDF pod-IVR, *P* = 0.3405; TDF-FTC pod-IVR, *P* = 0.5882. The median Nugent score prior to IVR placement (Visits 0 and 1) was 3.0 (IQR, 3.0–3.3) and with the TDF and TDF-FTC pod-IVRs in place was 2.5 (IQR, 2.0–4.0) and 4.5 (IQR, 2.8–5.3), respectively. There was no statistically significant difference in Nugent score prior to pod-IVR placement compared to pod-IVR: TDF pod-IVR, *P* = 0.6068; TDF-FTC pod-IVR, *P* = 0.5611.

The median vaginal pH prior to IVR placement (Visits 0 and 1) was 4.1 (IQR, 4.1.–4.5) and with the TDF-FTC-MVC pod-IVRs in place was 4.4 (IQR, 4.1–4.5). There was no statistically significant difference (0.3775) in vaginal pH prior to pod-IVR placement compared to during pod-IVR use. The median Nugent score prior to IVR placement (Visits 0 and 1) was 4.0 (IQR, 2.8–5.3) and with pod-IVRs in place was 5.0 (IQR, 4.0–5.0). These findings could be attributed to the prevalence of *Gardnerella vaginalis* in some of the participants’ VMBs (S3 Fig). There was no statistically significant difference (*P* = 0.5858) in Nugent score prior to pod-IVR placement compared to during pod-IVR.

### User perception and product acceptability

All participants in the TDF and TDF-FTC treatment periods were willing to recommend the ring to others, and 4 of 6 would “probably” or “definitely” use the pod-IVR for HIV prevention. The remaining 2 expressed no perceived risk of HIV acquisition. Participants’ confidence in their ability to insert and remove the IVR either held constant between using the first and second study rings or improved. Participants’ willingness to use the pod-IVR for 28 d and access the ring for “real world” use was high. For example, after using the first ring, participants averaged 3.5 (on a 1–5 Likert scale from “not at all confident” to “completely confident”) that she could use the ring for 28 d, while the average increased to 4.0 after using the second ring. Qualitative interview data revealed that, overall, the pod-IVRs were well tolerated by participants. Prior to insertion of the pod-IVR, clinicians reviewed verbal instructions with the participants, which reportedly allowed women to feel more confident in their ability to insert the pod-IVR on their own: clinician’s checking pod-IVR placement on first insertion also led to greater confidence in some. While 2 of 6 women felt the TDF-FTC pod-IVR was more difficult to insert than the TDF pod-IVR, the majority of participants felt both pod-IVRs were flexible and easy to insert. The majority of women noted that they did not feel the pod-IVR in their vaginas at all during the use period. Of note, psychological awareness of the pod-IVR was reported by one woman, which subsided after a few days. Lack of physical awareness of the pod-IVRs allowed most participants to continue their typical physical activity routines during the weeks of use, with one exception (i.e., an elite runner). All participants felt that the pod-IVRs had no impact on their typical hygiene practices. All participants were able to successfully remove the pod-IVRs, with 3 of 6 women reporting difficulty initially locating the TDF pod-IVRs in their vaginas.

All 6 women from the TDF-FTC-MVC pod-IVR study group reported that they would use the device if it were a 7-d HIV prevention ring; 5 of 6 said they would use it if it were a 28-d device. All 6 were willing to recommend the IVR to others. With respect to use behaviors, all reported being highly confident that they could insert the IVR correctly. The two participants who previously used the TDF and TDF-FTC pod-IVRs noted that inserting the TDF-FTC-MVC pod-IVR was much easier than the first two pod-IVRs they had used, because they were familiar with the insertion process and confident they could insert the device correctly. Of the 6 women who used the TDF-FTC-MVC pod-IVR, none reported concerns with being able to remove the device; ultimately, all thought removing it was easy and were able to do so successfully. When asked about preferred access should the IVR become available, 3 of the women would use the pod-IVR if accessed by prescription, and 4 would use the pod-IVR if accessed over the counter.

Qualitative interview data revealed that, overall, the TDF-FTC-MVC pod-IVR was well tolerated by participants. Prior to insertion of the pod-IVR, clinicians provided verbal instructions. Participants reported that their confidence to insert the pod-IVR was increased by the clinician’s instructions, as well as when the clinician checked for proper pod-IVR placement on first insertion. The majority (5 of 6) of the women noted that they did not feel the pod-IVR in their vaginas at all during the use period. None of the participants felt that the pod-IVRs had an impact on their typical hygiene practices. All 6 expressed the desire for the pod-IVR to have multiple purposes, such as for HIV prevention and contraception. Detailed data of user experiences are reported elsewhere [39].

### In vivo ARV drug release rates

In vivo release rate measurements are based on the residual drug mass remaining in the used IVRs and the assumption, supported by in vitro data [26,28,29], that drug release is linear over the period of IVR use. The mean daily in vivo IVR drug release rates in used IVRs were as follows: median (IQR); TDF pod-IVR; TDF, 0.81 (0.66–1.07) mg/d; TDF-FTC pod-IVR; TDF, 0.98 (0.88–1.93) mg/d; FTC, 1.99 (1.70–2.31) mg/d (S1 and S2 Tables). Importantly, >95% of the residual TDF in the used IVR pods was present as the prodrug by HPLC, i.e., no significant prodrug hydrolysis was observed following 1 wk of use in vivo. The in vivo release rates of TDF from the TDF and TDF-FTC pod-IVR were found to be not significantly different (*P* = 0.4286).

For in vivo release rates under 1 mg/d TDF, less than 4% of the IVR drug content would have been released over 1 wk of IVR use, making differential residual drug measurements challenging. The amount of TDF released in the TDF-FTC-MVC pod-IVR treatment period could not be quantified accurately, although it was higher than in the TDF and TDF-FTC pod-IVR treatment periods, based on median TDF CVF concentrations (S3 Table). The mean daily in vivo IVR drug release rates were as follows: median (IQR); FTC, 2.37 (1.94–2.57) mg/d; MVC, 2.07 (1.77–2.09) mg/d.

### Summary of drug concentration measurements

Drug and drug metabolite concentrations in key anatomic compartments with the IVRs in place are summarized in S1–S3 Tables. These data show that the IVRs maintained high ARV drug exposure in CVFs and vaginal tissues (VTs) relative to lower concomitant plasma concentrations. FTC and MVC drug concentrations in all matrices were higher than corresponding TDF/TFV concentrations as expected based on the residual drug levels in used IVRs.

### CVF drug levels

**Fig 1:**
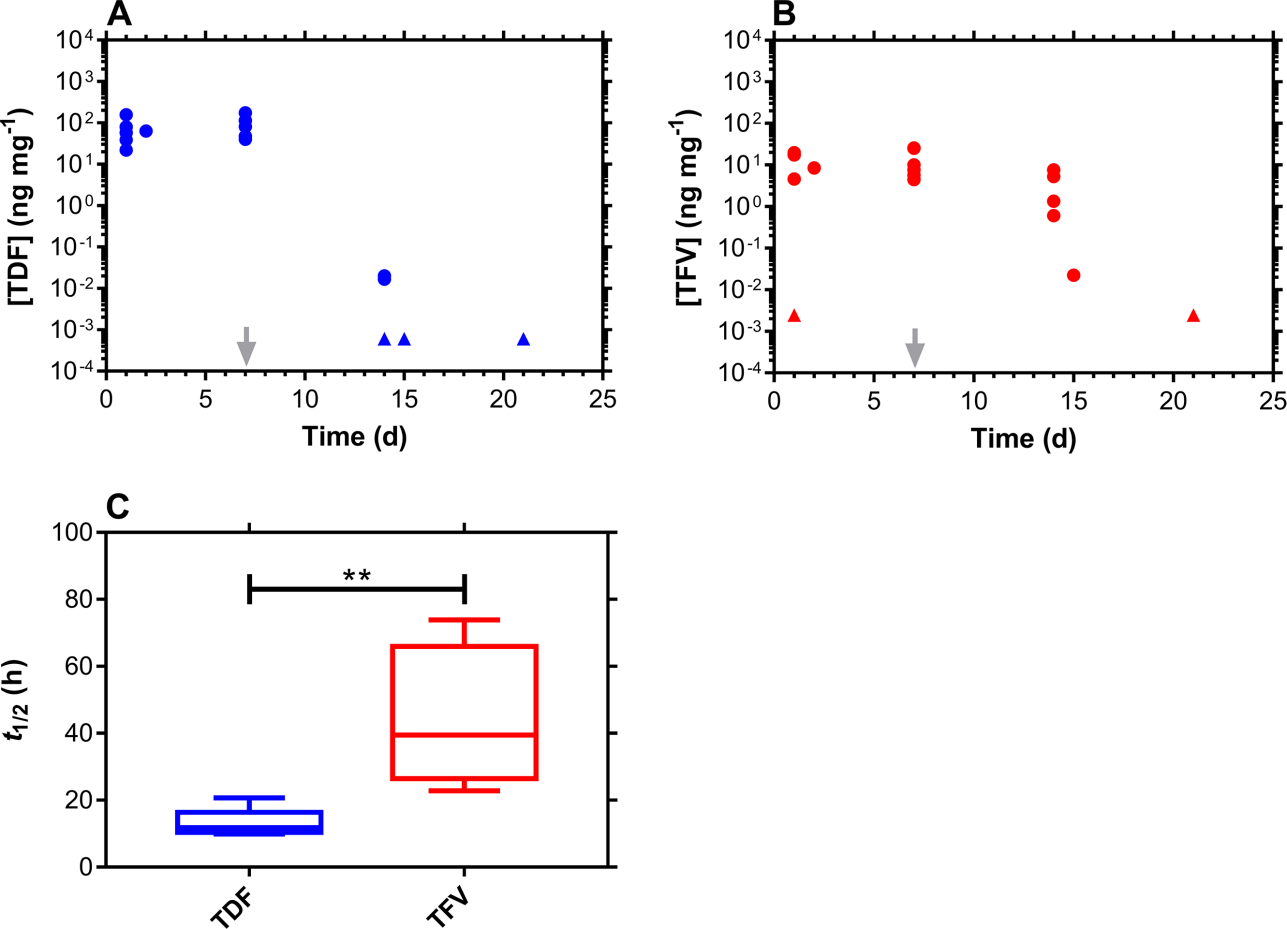
CVF drug levels and washout following TDF pod-IVR use. ARV drug levels in CVF fall off rapidly following IVR removal (grey arrow) but are quantifiable for a further 2 wk. Every circular datum represents an individual sample from one of the participants (*n* = 6), while triangles depict samples that were BLQ of the analytical method, and values were calculated as follows: [(assay BLQ)/2]/(median swab mass). (A) TDF. (B) TFV. (C) Box plots of ARV drug terminal half-lives of elimination from CVF. The box extends from the 25th to 75th percentiles, with the horizontal line in the box representing the median; whiskers represent the lowest and highest datum. Comparison of the TDF and TFV groups using an unpaired *t* test with Welch’s correction showed that the groups were significantly different (*P* = 0.0053). ARV, antiretroviral; BLQ, below the lower limit of quantitation; CVF, cervicovaginal fluid; IVR, intravaginal ring; TDF, tenofovir disoproxil fumarate; TFV, tenofovir.

**Fig 2:**
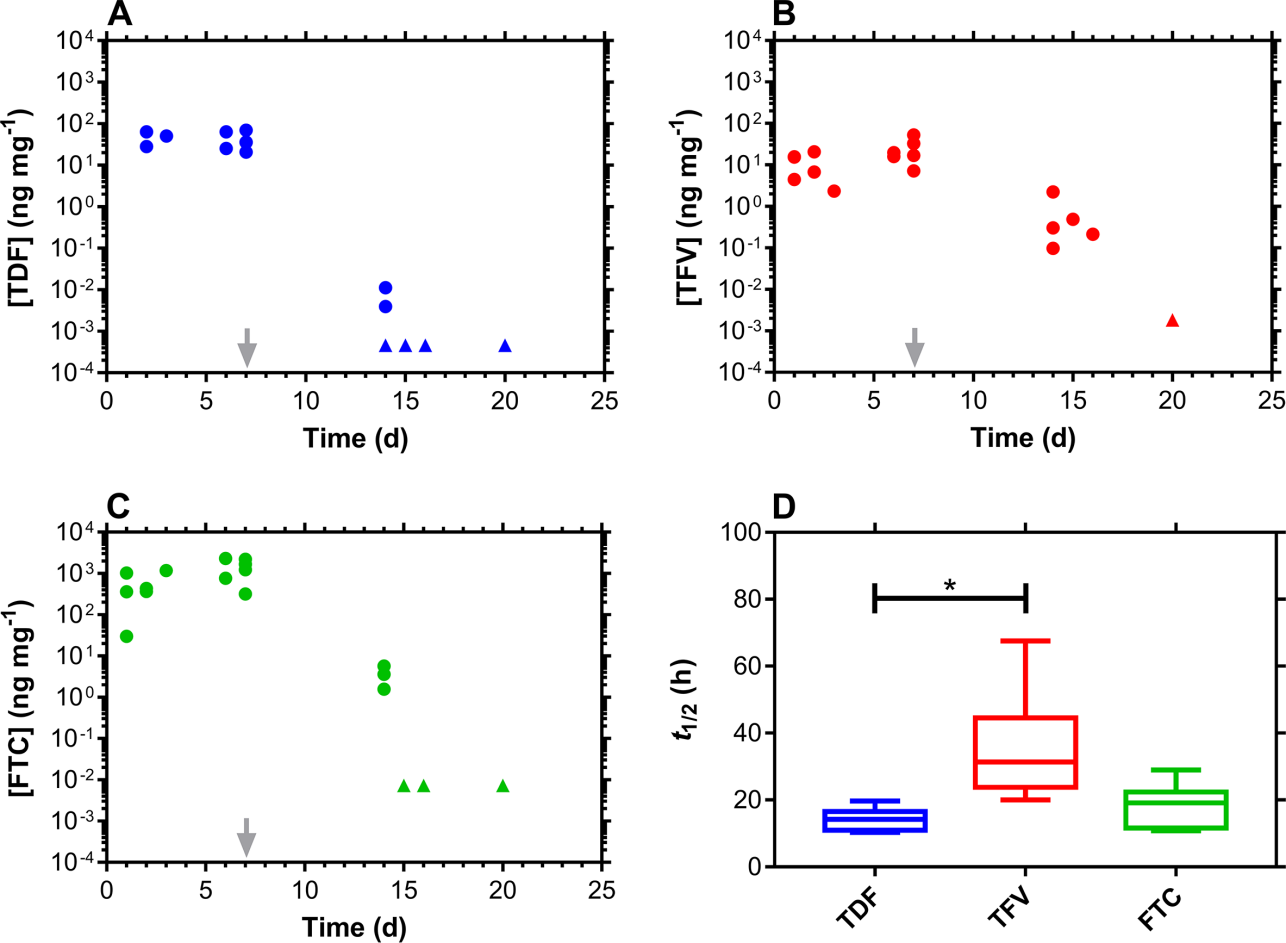
CVF drug levels and washout following TDF-FTC pod-IVR use. ARV drug levels in CVF fall off rapidly following IVR removal (grey arrow) but are quantifiable for a further 2 wk. Every circular datum represents an individual sample from one of the participants (*n* = 6), while triangles depict samples that were BLQ of the analytical method, and values were calculated as follows: [(assay BLQ)/2]/(median swab mass). (A) TDF. (B) TFV. (C) FTC. (D) Box plots of ARV drug terminal half-lives of elimination from CVF. The box extends from the 25th to 75th percentiles, with the horizontal line in the box representing the median; whiskers represent the lowest and highest datum. Comparison of the TDF and TFV groups using an unpaired *t* test with Welch’s correction showed that the groups were significantly different (*P* = 0.0131). ARV, antiretroviral; BLQ, below the lower limit of quantitation; CVF, cervicovaginal fluid; FTC, emtricitabine; IVR, intravaginal ring; TDF, tenofovir disoproxil fumarate; TFV, tenofovir.

**Fig 3:**
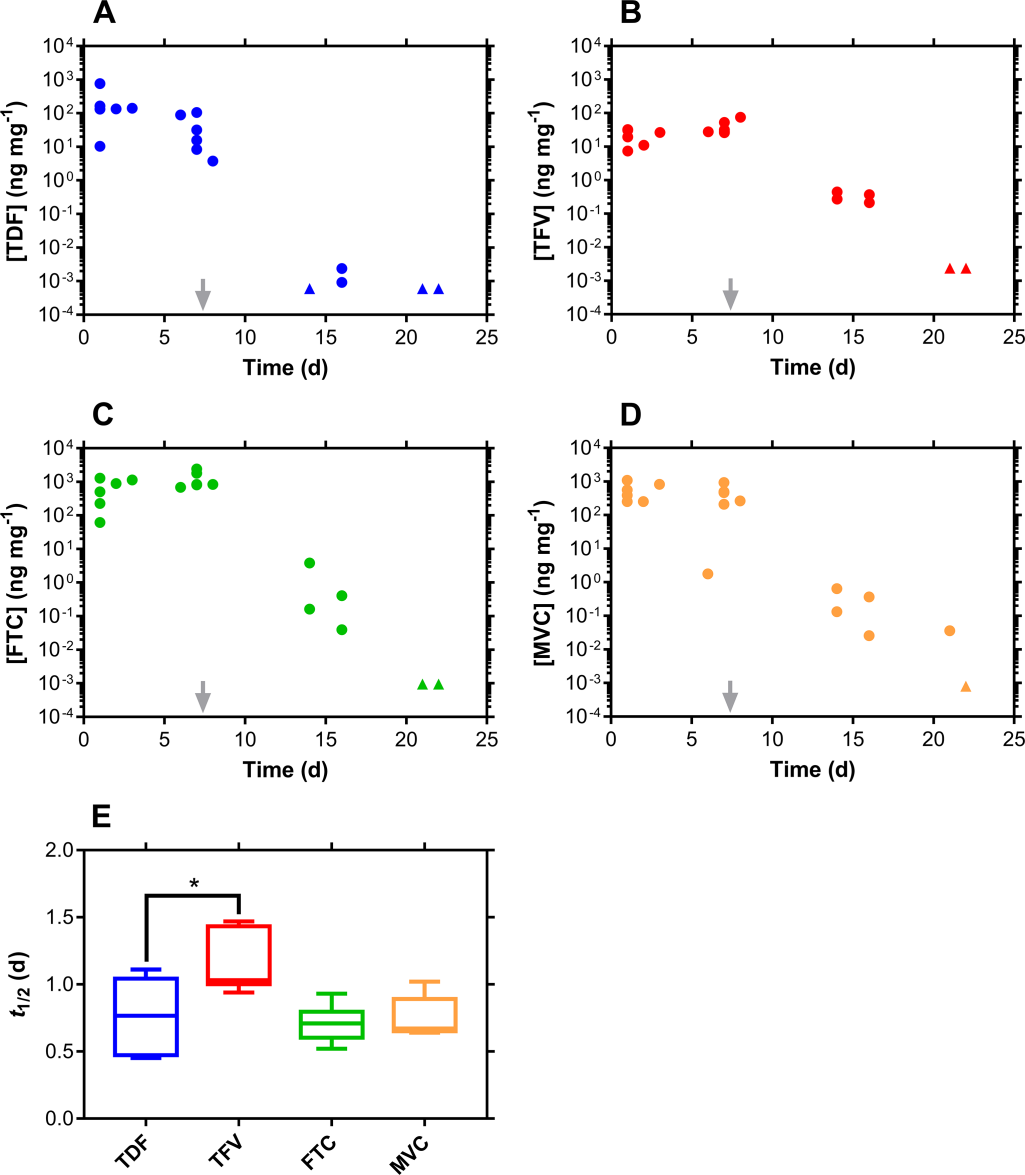
CVF drug levels and washout following TDF-FTC-MVC pod-IVR use. ARV drug levels in CVFs fall off rapidly following IVR removal (grey arrows) but are quantifiable for a further 2 wk. Every circular datum represents an individual sample from one of the participants (*n* = 6), while triangles depict samples that were BLQ of the analytical method, and values were calculated as follows: [(assay BLQ)/2]/(median swab mass). (A) TDF. (B) TFV. (C) FTC. (D) MVC. (E) Box plots of ARV drug terminal half-lives of elimination from CVF. The box extends from the 25th to 75th percentiles, with the horizontal line in the box representing the median; whiskers represent the lowest and highest datum. Comparison of the TDF, FTC, and MVC groups using an ordinary 1-way ANOVA analysis (no matching or pairing of the data) showed that they were not significantly different (*P* = 0.8973). However, comparison of the TDF and TFV groups using an unpaired *t* test with Welch’s correction, showed that the groups were significantly different (*P* = 0.0327). ANOVA, analysis of variance; ARV, antiretroviral; BLQ, below the lower limit of quantitation; CVF, cervicovaginal fluid; FTC, emtricitabine; IVR, intravaginal ring; MVC, maraviroc; TDF, tenofovir disoproxil fumarate; TFV, tenofovir.

CVF ARV drug concentrations all exhibited low variability during IVR use in all treatment periods (Figs 1–3), with the exception of one low data point at Visit 3 for MVC (Fig 3D). TDF CVF concentrations in the three groups (i.e., TDF, TDF-FTC, and TDF-FTC-MVC) were not statistically significantly different (*P* = 0.4233); however, TFV concentrations were different (*P* = 0.0118) with this test. Total TFV CVF concentrations, defined as the molar sum of paired TFV and TDF concentrations reported as TFV, across all three groups also were different (*P* = 0.0155), as detailed below, with higher median concentrations in the TDF-FTC-MVC pod-IVR group. FTC CVF concentrations in the TDF-FTC and TDF-FTC-MVC groups were not significantly different (*P* = 0.9323).

A comparison of the medians and IQRs shows that the corresponding TDF (S1 Table) and TDF-FTC (S2 Table) pod-IVR datasets are similar, but the TDF-FTC-MVC (S3 Table) pod-IVR leads a total (i.e., TDF + TFV, on a molar basis) TFV exposure that is ca. two times higher: median (IQR); **TDF**; TDF pod-IVR, 58.1 (43.9–97.4) ng/mg; TDF-FTC pod-IVR, 43.1 (31.0–65.0) ng/mg; TDF-FTC-MVC pod-IVR, 96.9 (14.5–137.1) ng/mg; **TFV**; TDF pod-IVR, 13.9 (6.2–19.3) ng/mg; TDF-FTC pod-IVR, 15.9 (7.1–20.0) ng/mg; TDF-FTC-MVC pod-IVR, 28.0 (24.3–31.9) ng/mg; **total TFV**; TDF pod-IVR, 36.2 (31.3–60.6) ng/mg; TDF-FTC pod-IVR, 34.4 (26.9–48.7) ng/mg; TDF-FTC-MVC pod-IVR, 70.0 (59.2–80.6) ng/mg.

The 2-wk window for the follow-up visit after IVR removal (Visit 4/8/4) allowed ARV drug washout kinetics to be measured and the corresponding terminal half-lives of elimination to be calculated (Figs 1–3): median (IQR); TDF pod-IVR; TDF, 11.8 (10.6–14.4) h; TFV, 39.5 (30.0–58.1) h; TDF-FTC pod-IVR; TDF, 14.2 (11.7–15.4) h; TFV, 31.4 (25.9–36.2) h; FTC, 19.1 (13.5–20.1) h; TDF-FTC-MVC pod-IVR; TDF, 18.3 (11.8–24.2) h; TFV, 24.8 (24.4–31.9) h; FTC, 17.0 (15.5–17.8) h; MVC, 16.0 (15.8–18.3) h. Comparison of the TDF and TFV half-lives in the TDF pod-IVR group showed that the datasets were different (*P* = 0.0043). The TDF and TFV half-lives in the TDF-FTC pod-IVR group also were different (*P* = 0.0022), but the TDF and FTC half-lives were not different (*P* = 0.2403). The ARV drug half-lives across IVR groups were not significantly different (TDF, *P* = 0.4848; TFV, *P* = 0.4286). Comparison of the TDF, FTC, and MVC groups (Fig 3) showed that they were not significantly different (*P* = 0.9320). However, comparison of just the TDF and TFV groups showed that they were significantly different (*P* = 0.0909).

The measurement of Li^+^, an exogenous tracer added to the naïve CVL fluid, was used to correct the CVL drug concentration analyses for dilution in the TDF-FTC-MVC pod-IVR group and, hence, affords the corresponding drug concentrations in undiluted CVF [37]. The corrected CVL drug concentrations exhibited a moderate (total TFV and MVC) to weak (FTC) correlation with paired CVF (Dacron swab) drug concentrations (S4 Fig): TFV (total TFV, reported as the molar sum of TDF and TFV concentrations); slope, 7.53 ± 0.84; *R*^*2*^, 0.889; FTC; slope, 2.40 ± 1.43; *R*^*2*^, 0.220; MVC; slope, 4.37 ± 1.11; *R*^*2*^, 0.609. The CVF volume collected during the lavage procedure (S4 Fig) with the TDF-FTC-MVC pod-IVRs in place was: median (IQR), 85.6 (28.0–116) μL.

### VT drug concentrations

**Fig 4:**
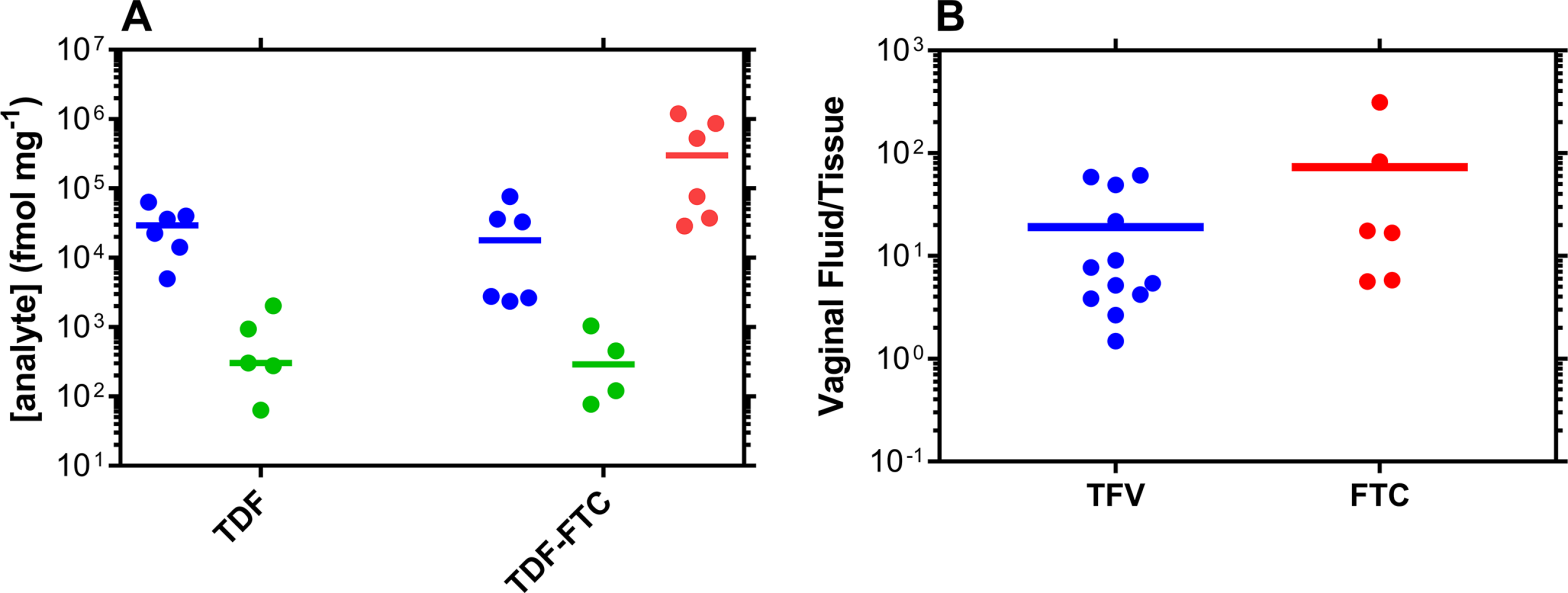
Drug and drug metabolite exposure in VT at TDF and TDF-FTC pod-IVR removal (Day 7). (A) ARV drug and TFV-DP levels in vaginal biopsies collected on Day 7 within 30 min of pod-IVR removal. Analyte concentrations are all expressed as fmol/mg of tissue homogenate to allow meaningful cross-comparison. Every circular datum represents an individual sample from one of the participants (*n* = 6); horizontal lines represent group medians; blue, TFV; green, TFV-DP; red, FTC. (B) Paired CVF:VT concentration ratios of TFV (molar sum of TDF and TFV concentrations) and FTC at Day 7; horizontal lines represent group means. The ratios provide a measure of the extent of tissue penetration for each analyte following vaginal delivery and, hence, vaginal bioavailability. The CVF TFV concentrations used in this calculation were the molar sum of the measured TFV and TDF concentrations as the prodrug hydrolyzes to TFV in the vaginal mucosa. ARV, antiretroviral; CVF, cervicovaginal fluid; FTC, emtricitabine; IVR, intravaginal ring; TDF, tenofovir disoproxil fumarate; TFV, tenofovir; TFV-DP, tenofovir diphosphate; VT, vaginal tissue.

**Fig 5:**
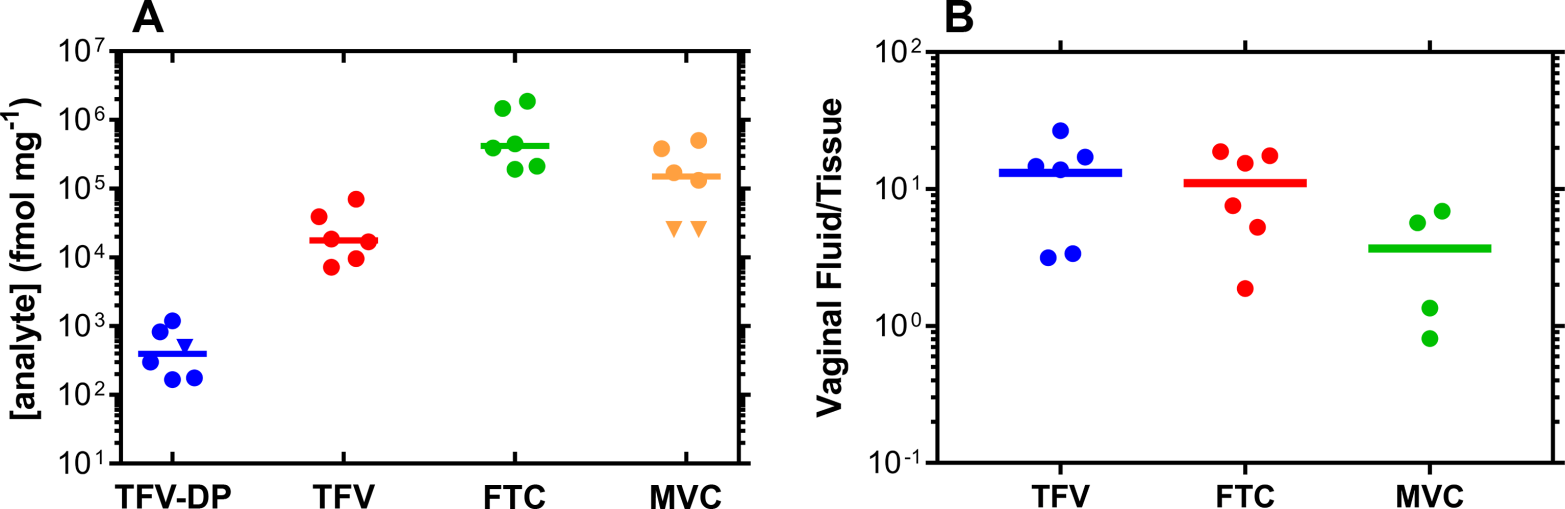
Drug and drug metabolite exposure in VTs at TDF-FTC-MVC pod-IVR removal (Day 7). (A) ARV drug and TFV-DP levels in vaginal biopsies collected on Day 7 within 30 min of pod-IVR removal. Analyte concentrations are all expressed as fmol/mg of tissue homogenate to allow meaningful cross-comparison. Every circular datum represents an individual sample from one of the participants (*n* = 6), while inverted triangles depict samples that were ALQ of the analytical method, and values were calculated as follows: (assay ALQ)/(median biopsy mass); horizontal lines represent group medians. (B) Paired CVF:VT concentration ratios of total TFV (molar sum of TDF and TFV concentrations), FTC, and MVC at Day 7; horizontal lines represent group means. The ratios describe the extent of tissue penetration for each analyte following vaginal delivery and, hence, vaginal bioavailability. The CVF TFV concentrations used in this calculation were the molar sum of the measured TFV and TDF concentrations, as the prodrug hydrolyzes to TFV in the vaginal mucosa. The two MVC VT samples that were ALQ were omitted from the analysis. There was no statistically significant difference between the three groups according to an unpaired, nonparametric Kruskal–Wallis test (*P* = 0.1591). ALQ, above the upper limit of quantitation; ARV, antiretroviral; CVF, cervicovaginal fluid; FTC, emtricitabine; IVR, intravaginal ring; MVC, maraviroc; TDF, tenofovir disoproxil fumarate; TFV, tenofovir; TFV-DP, tenofovir diphosphate; VT, vaginal tissue.

Molar antiviral drug concentrations in VT biopsy homogenate are described in Fig 4A and Fig 5A to allow direct comparison with the pharmacologically active metabolite of TFV against HIV, TFV-DP. The CVF (ng/mg) to VT (ng/mg) drug concentration ratio (Fig 4B and Fig 5B) provides a simple measure of xenobiotic partitioning between the two anatomic compartments: the lower the ratio, the more the antiviral agent distributes into the vaginal mucosa and the higher the vaginal bioavailability. Drug CVF:VT median (IQR) ratios are as follows: TDF and TDF-FTC pod-IVRs (data combined); TFV (molar sum of TDF and TFV, as TFV), 6.6 (4.1–28.6); FTC, 17.2 (8.5–66.5). The concentration ratio for TDF is 2.6 times lower than for FTC; TDF-FTC-MVC pod-IVR; TFV, 14.2 (6.0–16.5); FTC, 11.5 (5.8–17.0); MVC 3.5 (0.9–6.6). There was no statistically significant difference between the three groups (*P* = 0.1591).

## Discussion

To our knowledge, the clinical trial described here is the first to evaluate long-acting vaginal delivery of TDF-FTC, the only FDA-approved drug regimen (in the oral formulation Truvada) for HIV PrEP [12]. The innovative open-label, crossover trial format allowed two IVR formulations—TDF alone and in combination with FTC—to be evaluated sequentially with each participant acting as her own control. To our knowledge, the trial also involved the first triple ARV combination IVR to be evaluated clinically. The pod-IVRs were safe and generally well tolerated, despite high VT ARV concentrations especially for FTC and MVC (Fig 5A). The implications of our findings are discussed below in the context of developing a viable HIV PrEP candidate targeted at resource-poor regions.

The 3 pod-IVRs maintained high, controlled ARV drug concentrations in CVF over the period of product use, while leading to low systemic exposures (S1–S3 Tables). The low plasma ARV drug concentrations are a benefit of topical dosing as the risks of systemic toxicity and the emergence of drug resistance are reduced. The CVF drug levels decreased sharply post-IVR removal on Day 7 (Figs 1–3). The drug washout profiles allowed the CVF half-lives of the three drugs as well as TFV, the hydrolysis product of TDF, to be measured for the first time (Fig 3E). In all three groups, TFV had a significantly longer half-life (median, 24.8–39.5 h) than the other agents. We previously have described a mechanism in sheep [40] in which TDF, delivered via IVR, distributed efficiently from the CVF into the VT, where it hydrolyzed enzymatically to TFV. The accumulation of TFV in the VT formed a depot that could release TFV back into the lumen. A similar mechanism may operate here in women, accounting for the longer TFV half-life in CVF relative to the other agents.

The systematically higher (2.4–7.5-fold) CVF drug concentrations collected via lavage versus Dacron swab in the TDF-FTC-MVC pod-IVR group was unexpected and remains largely unexplained. It is possible that the lavage is more efficient at extracting drug that is associated with the surface of the vaginal epithelium. While we have used the Li^+^ tracer technique previously to measure CVF dilution in CVL in sheep [29], this is the first example of its application in a clinical setting for PK analysis. During IVR use, the median CVF volumes (0.086 mL; IQR, 0.028–0.12 mL) in the 6 participants were lower than the volumes collected previously in women at different phases of the menstrual cycle, 0.30 ± 0.22 mL (follicular phase) and 0.45 ± 0.21 mL (luteal phase), using a different assay [41]. This was expected based on the collection of multiple samples (one swab for VMB analysis, one pH swab, two Dacron swabs for CVF drug analyses, and one vaginal sidewall scraping for microbial biofilm imaging) prior to the lavage. As a result, a significant fraction of the available CVF was removed before CVL collection.

In HIV PrEP, there is no biomarker of ARV drug effect in susceptible, uninfected individuals to guide product development, unlike treatment of HIV-1/AIDS. The choice of ARV agent and target drug levels in key pharmacological compartments largely is based on theoretical arguments and results from preclinical studies. The strategy employed here was based on using FDA-approved ARV drugs that have shown clinical efficacy in HIV PrEP using oral or topical regimens [4,5,7–11,42]. Combined with PK data from oral dosing randomized clinical trials demonstrating efficacy [7,42], one can bracket the concentration targets associated with vaginal protection. A randomized, PK, cross-over study (MTN-001) compared TFV vaginal gel and oral TDF tablets by measuring drug and drug metabolite levels in VT from 144 HIV-uninfected women [32]. The TFV concentrations in VT homogenate at end-of-period visit were as follows: median (IQR); vaginal TFV gel, 113 (27–265) ng/mg; oral TDF, 0.15 (0.15–0.27) ng/mg. Assuming that the range of drug concentrations obtained from these dosing modalities is a key determinant for efficacy in preventing vaginal acquisition of HIV, the TFV concentrations in vaginal biopsies collected on Day 7 at IVR removal in this study, 8.4 (4.7–11.2) ng/mg (S1 Table), are suggestive of positive pharmacodynamic outcomes. However, this analysis assumes oral dosing is not fundamentally different than topical dosing in terms of tissue concentrations required for high PrEP efficacy.

A previous clinical trial evaluating a different TDF IVR platform—a reservoir IVR, in which a solid TDF formulation is contained in a hollow hydrophilic polyether urethane tube and the drug is delivered through the ring elastomer—reported median CVF TDF and TFV concentrations during ring use of 110 ng/mg and 70 ng/mg, respectively [43]. These levels are higher than the corresponding drug concentrations measured here (S1–S3 Tables), although the TDF:TFV ratio was considerably higher in our study (2.7–4.2-fold, depending on the formulation, compared to 1.5) and the TDF CVF concentrations were more stable (Figs 1–2). Together, these results suggest less TDF hydrolysis either in the IVRs during use or sample handling in our trial. Sample bioanalysis in both trials was carried out by the same laboratory using the same methods. The observation may be significant since the VT bioavailability in sheep of the TDF prodrug is nearly 100 times higher than for parent TFV [40].

Immune cells in the vaginal mucosa are believed to be the key pharmacologic compartment determining the efficacy of vaginal HIV PrEP. It is not feasible in early-stage clinical trials to collect sufficient VT to extract immune cells for intracellular drug analysis. VT biopsy homogenate has been shown to correlate well with CD4^+^ cells extracted from VT [44], and the relevant analytes are TFV, TFV-DP, the pharmacologically active metabolite of TFV against HIV, and FTC (Figs 4A and 5A). Due to the small amount of VT collected, the active, triphosphorylated metabolite of FTC could not be measured here. Median VT homogenate TFV-DP concentrations of 303 (TDF pod-IVR), 289 (TDF-FTC pod-IVR), and 302 (TDF-FTC-MVC pod-IVR) fmol/mg were measured post-IVR removal. These concentrations are 2–3-fold higher than the 120 fmol/mg median ectocervical biopsy levels obtained with a TDF reservoir IVR in a recent clinical trial [43]. Importantly, the median TFV and FTC VT concentrations (TDF pod-IVR; TFV, 8.4 ng/mg; TDF-FTC pod-IVR; TFV, 5.1 ng/mg; FTC, 75 ng/mg) were lower than those obtained in pigtailed macaques (TFV 28–35 and FTC 460–650 ng/mg, depending on the sampling location) with TDF-FTC pod-IVRs [26], which provided complete protection from Simian Human Immunodeficiency Virus (SHIV) infection in pigtailed macaques using the rigorous, repeat low-dose challenge model [45]. Smith and colleagues used the same model to evaluate a reservoir TDF IVR and also obtained complete protection from SHIV infection [46]. The median VT TFV concentration around 10 ng/mg observed with these reservoir TDF IVRs is comparable to the VT TFV values obtained in our study. When complemented by ca. 10 times higher tissue FTC levels, as observed here, these results suggest that the devices may be effective in HIV PrEP.

No clinical efficacy data for HIV PrEP using intravaginal MVC currently are available, and the levels in the pharmacologically relevant compartments required to afford protection are, therefore, unknown. The published in vitro antiviral potencies of MVC against HIV-1 primary and laboratory-adapted isolates in peripheral blood mononuclear cells (PBMCs) span a wide range of inhibitory concentrations: IC_50_, 0.1–4.5 nM; IC_90_, 0.5–13.4 nM [47]. The observed median MVC concentrations of 142 ng/mg (276 μM) in VTs on Day 7 were more than 20,000 times higher than the highest IC_90_, suggesting favorable pharmacodynamic outcomes. A randomized clinical trial (MTN-013/IPM 026) in 48 HIV-negative women evaluating matrix-IVRs delivering MVC alone or in combination with DPV measured MVC CVF concentrations of 2.5 and 1.1 ng/mg, respectively, at Day 28 when the IVRs were removed [48]. These concentrations are 170–390 times lower than those obtained in the current study (S3 Table). It should be noted that matrix-IVRs tend to have a drug release burst in the first week. VT MVC levels in MTN013 were below the lower limit of quantification for all subjects using the DPV-MVC IVR and were only quantifiable in 4 of the 12 MVC IVR users, with a 0.13–4.4 ng/mg concentration range, much lower than the median concentration (142 ng/mg) measured following pod-IVR delivery reported here (S3 Table).

To our knowledge, the ASPIRE study and The Ring Study were the first published Phase 3 studies of an ARV (i.e., DPV) delivered via IVR for HIV PrEP [49,50]. In the ASPIRE study, the incidence of HIV infection in the DPV group was 27% lower than in the placebo group; this improved to 56% protection in women over the age of 21 years; the difference was attributed to better adherence in women over the age of 21 years [49]. Both studies found low rates of adherence, particularly in young women raising concerns about the viability of IVRs for HIV PrEP in resource-limited regions like sub-Saharan Africa, where contraceptive IVRs are not as commonly used as in the developed world. It is believed by some that with increased familiarity with IVRs, adherence to IVR use will improve [51–55] and make IVR-based ARV regimens viable. This belief is based on previous experience with oral HIV PrEP regimens that demonstrated a dramatic increase in adherence when moving from the initial, blinded, placebo-controlled trials to the open-label follow-on trials [11,56–58].

In terms of the user perception and acceptability data, the pod-IVR delivery device was both easy to use and well tolerated. Women were willing to use it within the context of this cross-over study and anticipated being confident in their ability to use it for longer periods of time in “real-world” settings for HIV prevention. While, on average, participant confidence in their ability to access and use the IVR stayed the same or increased between uses, one participant’s data suggest that her confidence in her ability to insert and remove the IVR decreased with her second experience, indicating that further study is necessary to determine the factors related to confidence and what is needed to become skilled in IVR insertion and removal. Women were willing to use the TDF-FTC-MVC pod-IVRs effectively within the context of the study and anticipated feeling confident in their ability to use it for HIV prevention for sustained periods of use. Additionally, given the design of the current study, future work will need to assess user perceptions and experiences of pod-IVRs for longer periods of use, as well as in relation to sexual activity and use during menstruation.

Limitations of this study are as follows. Women at low risk for acquisition of HIV were recruited for this study since it was a first in humans and was primarily focused on PK and safety; these women may have different perceptions about HIV prevention than women at high risk for HIV, who will be the targeted population for these prevention products. The small sample size was also a limitation; however, the primary goal of the study was to show drug release and initial safety, as well as to get initial understanding of women’s perceptions and acceptability of the IVRs. The short duration was also a limitation since the IVRs will be used for 28 d; however, this initial study of 7 d duration was required by the FDA prior to a 28-d study. A 28-d study is planned as a follow-on to this study. Lastly, as described above, due to the small size of the vaginal biopsies, we were unable to measure FTC triphosphate in VT.

In conclusion, a crossover Phase I clinical trial sequentially evaluated TDF, TDF-FTC, and TDF-FTC-MVC pod-IVRs and demonstrated that the devices exhibited favorable safety and PK profiles across all three treatment periods. The results justify longer and larger follow-on clinical trials in the future.

## Supporting information

S1 TREND Checklist TREND Checklist with associated paragraph numbers. (PDF)

S1 Fig Microbial community profiles for the six trial participants in the TDF pod-IVR trial arm determined by a custom qPCR array. The IVRs were inserted at V1 and removed at V3. IVR, intravaginal ring; qPCR, quantitative polymerase chain reaction; TDF, tenofovir disoproxil fumarate.
(TIF)

S2 Fig Microbial community profiles for the six trial participants in the TDF-FTC pod-IVR trial arm determined by a custom qPCR array. The IVRs were inserted at V5 and removed at V7. FTC, emtricitabine; IVR, intravaginal ring; qPCR, quantitative polymerase chain reaction; TDF, tenofovir disoproxil fumarate.
(TIF)

S3 Fig Microbial community profiles for the six trial participants in the TDF-FTC-MVC pod-IVR trial arm determined by a custom qPCR array. The IVRs were inserted at V1 and removed at V3. FTC, emtricitabine; IVR, intravaginal ring; MVC, maraviroc; qPCR, quantitative polymerase chain reaction; TDF, tenofovir disoproxil fumarate.
(TIF)

S4 Fig Measurement of CVF volume collected in the lavage procedure allows for dilution correction of the CVL analyses. Drug concentrations with the IVR in place (V2–V3) in CVL samples corrected for CVF dilution (y-axis) plotted against paired drug concentrations in neat CVF samples collected using Dacron swabs (x-axis) exhibit a moderate-weak correlation, with systematically higher values in CVL samples. (A) TFV (total TFV, reported as the molar sum of TDF and TFV concentrations in the samples); slope, 7.53 ± 0.84; R2, 0.889; FTC; slope, 2.40 ± 1.43; R2, 0.220; MVC; slope, 4.37 ± 1.11; *R*^*2*^, 0.609. (D) Box plots of CVF volume collected for all participants (*n* = 6) at each study visit (V1–V4). The box extends from the 25th to 75th percentiles, with the horizontal line in the box representing the median; whiskers represent the lowest and highest datum. CVF, cervicovaginal fluid; CVL, cervicovaginal lavage; FTC, emtricitabine; IVR, intravaginal ring; MVC, maraviroc; TDF, tenofovir disoproxil fumarate; TFV, tenofovir.
(TIF)

S1 Table Summary of drug and drug metabolite concentrations in key anatomic compartments measured with TDF pod-IVR in place (6 participants), i.e., Visits 2 and 3. IVR, intravaginal ring; TDF, tenofovir disoproxil fumarate.
(DOCX)

S2 Table Summary of drug and drug metabolite concentrations in key anatomic compartments measured with TDF-FTC pod-IVR in place (6 participants), i.e., Visits 6 and 7. FTC, emtricitabine; IVR, intravaginal ring; TDF, tenofovir disoproxil fumarate.
(DOCX)

S3 Table Summary of drug and drug metabolite concentrations in key anatomic compartments measured with TDF-FTC-MVC pod-IVR in place (6 participants), i.e., Visits 2 and 3. FTC, emtricitabine; IVR, intravaginal ring; MVC, maraviroc; TDF, tenofovir disoproxil fumarate.
(DOCX)

S1 Protocol Summary of clinical trial protocol. (DOCX)

## Abbreviations

AE: adverse event
ALQ: above the upper limit of quantitation
ARV: antiretroviral
ASPIRE: A Study to Prevent Infection with a Ring for Extended Use
BLQ: below the lower limit of quantitation
CAPRISA: Centre for the AIDS Programme of Research in South Africa
CASI: computer-assisted self-interview
CDC: Centers for Disease Control
CVF: cervicovaginal fluid
CVL: cervicovaginal lavage
DMF: Drug Master File
DPV: dapivirine
FACTS: Follow-on African Consortium for Tenofovir Studies
FDA: Food and Drug Administration
FTC: emtricitabine
HPLC: high-performance liquid chromatography
IPM: International Partnership for Microbicides
IVR: intravaginal ring
KOH: potassium hydroxide
LC-MS/MS: liquid chromatographic-tandem mass spectrometry
LLOQ: lower limit of quantification
MTN: Microbicide Trials Network
MVC: maraviroc
NCA: noncompartmental analysis
NRTI: nucleoside reverse transcriptase inhibitor
PBMC: peripheral blood mononuclear cell
PDMS: polydimethylsiloxane
PK: pharmacokinetic
PrEP: pre-exposure prophylaxis
qPCR: quantitative polymerase chain reaction
SAE: serious adverse event
SHIV: Simian Human Immunodeficiency Virus
STI: sexually transmitted infection
TDF: tenofovir disoproxil fumarate
TFV: tenofovir
TFV-DP: tenofovir diphosphate
UNAIDS: Joint United Nations Programme on HIV/AIDS
USPE: user sensory perception and experience
VMB: vaginal microbiome
VOICE: Vaginal and Oral Interventions to Control the Epidemic
VT: vaginal tissue

## References

[1] Birx DL. Delivering an AIDS-free generation [Presentation]: The Henry J. Kaiser Family Foundation; 2014.

[2] Fast-Track: Ending the AIDS Epidemic by 2030. Geneva, Switzerland: UNAIDS, 2014.

[3] Prevention Gap Report. Geneva, Switzerland: UNAIDS, 2016.

[4] Abdool Karim Q, Abdool Karim SS, Frohlich JA, Grobler AC, Baxter C, Mansoor LE, et al Effectiveness and Safety of Tenofovir Gel, an Antiretroviral Microbicide, for the Prevention of HIV Infection in Women. Science. 2010;329(5996):1168–74. 10.1126/science.1193748 20643915 PMC3001187

[5] Grant RM, Lama JR, Anderson PL, McMahan V, Liu AY, Vargas L, et al Preexposure Chemoprophylaxis for HIV Prevention in Men Who Have Sex with Men. N Engl J Med. 2010;363(27):2587–99. PubMed PMID: WOS:000285763700004. 10.1056/NEJMoa1011205 21091279 PMC3079639

[6] Baeten JM, Donnell D, Ndase P, Mugo NR, Campbell JD, Wangisi J, et al Antiretroviral prophylaxis for HIV prevention in heterosexual men and women. N Engl J Med. 2012;367(5):399–410. 10.1056/NEJMoa1108524 ; PubMed Central PMCID: PMC3770474. 22784037 PMC3770474

[7] Thigpen MC, Kebaabetswe PM, Paxton LA, Smith DK, Rose CE, Segolodi TM, et al Antiretroviral Preexposure Prophylaxis for Heterosexual HIV Transmission in Botswana. N Engl J Med. 2012;367(5):423–34. 10.1056/NEJMoa1110711 PubMed PMID: WOS:000307001900007. 22784038

[8] Choopanya K, Martin M, Suntharasamai P, Sangkum U, Mock PA, Leethochawalit M, et al Antiretroviral Prophylaxis for HIV Infection in Injecting Drug Users in Bangkok, Thailand (the Bangkok Tenofovir Study): a Randomised, Double-blind, Placebo-controlled Phase 3 Trial. Lancet. 2013;381(9883):2083–90. 10.1016/S0140-6736(13)61127-7 23769234

[9] Molina JM, Capitant C, Spire B, Pialoux G, Cotte L, Charreau I, et al On-demand Preexposure Prophylaxis in Men at High Risk for HIV-1 Infection. N Engl J Med. 2015;373(23):2237–46. 10.1056/NEJMoa1506273 PubMed PMID: WOS:000365743100007. 26624850

[10] Marcus JL, Hurley LB, Hare CB, Nguyen DP, Phengrasamy T, Silverberg MJ, et al Preexposure Prophylaxis for HIV Prevention in a Large Integrated Health Care System: Adherence, Renal Safety, and Discontinuation. J Acquir Immune Defic Syndr. 2016;73(5):540–6. PubMed PMID: WOS:000389045300011. 10.1097/QAI.0000000000001129 27851714 PMC5424697

[11] McCormack S, Dunn DT, Desai M, Dolling DI, Gafos M, Gilson R, et al Pre-exposure Prophylaxis to Prevent the Acquisition of HIV-1 Infection (PROUD): Effectiveness Results from the Pilot Phase of a Pragmatic Open-label Randomised Trial. Lancet. 2016;387(10013):53–60. 10.1016/S0140-6736(15)00056-2 PubMed PMID: WOS:000367457300027. 26364263 PMC4700047

[12] Roehr B. FDA Approves First Drug to Prevent HIV Infection. B M J. 2012;345:e4879. 10.1136/bmj.e4879 22807165

[13] Sheth AN, Rolle CP, Gandhi M. HIV Pre-exposure Prophylaxis for Women. J Virus Erad. 2(3):149–55. 27482454 10.1016/S2055-6640(20)30458-1 PMC4967966

[14] Amico KR, Mansoor LE, Corneli A, Torjesen K, van der Straten A. Adherence Support Approaches in Biomedical HIV Prevention Trials: Experiences, Insights and Future Directions from Four Multisite Prevention Trials. AIDS Behav. 2013;17(6):2143–55. 10.1007/s10461-013-0429-9 23435697 PMC3672509

[15] Kruse W, Eggertkruse W, Rampmaier J, Runnebaum B, Weber E. Dosage Frequency and Drug Compliance Behavior—a Comparative Study on Compliance with a Medication to Be Taken Twice or 4 Times Daily. Eur J Clin Pharmacol. 1991;41(6):589–92. 10.1007/BF00314990 PubMed PMID: WOS:A1991GU48100016. 1815972

[16] Sershen S, West J. Implantable, Polymeric Systems for Modulated Drug Delivery. Adv Drug Deliv Rev. 2002;54(9):1225–35. 10.1016/s0169-409x(02)00090-x PubMed PMID: WOS:000179294000006. 12393303

[17] Kutilek VD, Sheeter DA, Elder JH, Torbett BE. Is Resistance Futile? Curr Drug Targets Infect Disord. 2003;3(4)::295–309. 14754431 10.2174/1568005033481079

[18] Yeaw J, Benner JS, Walt JG, Sian S, Smith DB. Comparing Adherence and Persistence Across 6 Chronic Medication Classes. J Manag Care Pharm. 2009;15(9):728–40. PubMed PMID: WOS:000272878200001. doi: 10.18553/jmcp.2009.15.9.728 19954264 10.18553/jmcp.2009.15.9.728 PMC10441195

[19] Moss JA, Baum MM. Microbicide Vaginal Rings In: das Neves J, Sarmento B, editors. Drug Delivery and Development of Anti-HIV Microbicides. Singapore: Pan Stanford Publishing; 2014 p. 221–90.

[20] McGowan I. Injectable and Implantable Antiretroviral Strategies for HIV Prevention. Future Virol. 2015;10(10):1163–76. PubMed PMID: WOS:000364917600006.

[21] Baeten JM, Palanee-Phillips T, Brown ER, Schwartz K, Soto-Torres LE, Govender V, et al Use of a Vaginal Ring Containing Dapivirine for HIV-1 Prevention in Women. N Engl J Med. 2016;375:2121–32. 10.1056/NEJMoa1506110 26900902 PMC4993693

[22] Nel A, van Niekerk N, Kapiga S, Bekker LG, Gama C, Gill K, et al Safety and Efficacy of a Dapivirine Vaginal Ring for HIV Prevention in Women. N Engl J Med. 2016;375(22):2133–43. 10.1056/NEJMoa1602046 PubMed PMID: WOS:000389030400005. 27959766

[23] Guthrie KM, Vargas S, Shaw JG, Rosen RK, van den Berg JJ, Kiser PF, et al The Promise of Intravaginal Rings for Prevention: User Perceptions of Biomechanical Properties and Implications for Prevention Product Development. PLoS ONE. 2015;10(12):e0145642 10.1371/journal.pone.0145642 26695431 PMC4690611

[24] Rosen RK, van den Berg JJ, Vargas SE, Senocak N, Shaw JG, Buckheit RW, et al Meaning-making Matters in Product Design: Users' Sensory Perceptions and Experience Evaluations of Long-acting Vaginal Gels and Intravaginal Rings. Contraception. 2015;92(6):596–601. 10.1016/j.contraception.2015.08.007 PubMed PMID: WOS:000365556500015. 26276246 PMC4664151

[25] Baum MM, Butkyavichene I, Gilman J, Kennedy S, Kopin E, Malone AM, et al An Intravaginal Ring for the Simultaneous Delivery of Multiple Drugs. J Pharm Sci. 2012;101(8):2833–43. PubMed Central PMCID: PMC3857731. 10.1002/jps.23208 22619076 PMC3857731

[26] Moss JA, Srinivasan P, Smith TJ, Butkyavichene I, Lopez G, Brooks AA, et al Pharmacokinetics and Preliminary Safety Study of Pod-Intravaginal Rings Delivering Antiretroviral Combinations for HIV Prophylaxis in a Macaque Model. Antimicrob Agents Chemother. 2014;58(9):5125–35. PubMed Central PMCID: PMC4135875. 10.1128/AAC.02871-14 24936594 PMC4135875

[27] Smith JM, Moss JA, Srinivasan P, Butkyavichene I, Gunawardana M, Fanter R, et al Novel Multipurpose Pod-intravaginal Ring for the Prevention of HIV, HSV, and Unintended Pregnancy: Pharmacokinetic Evaluation in a Macaque Model. PLoS ONE. 2017;12(10):e0185946 10.1371/journal.pone.0185946 28982161 PMC5628903

[28] Baum MM, Butkyavichene I, Churchman SA, Lopez G, Miller CS, Smith TJ, et al An Intravaginal Ring for the Sustained Delivery of Tenofovir Disoproxil Fumarate. Int J Pharm. 2015;495(1):579–87. PubMed Central PMCID: PMC4609628. 10.1016/j.ijpharm.2015.09.028 26386138 PMC4609628

[29] Moss JA, Butkyavichene I, Churchman SA, Gunawardana M, Fanter R, Miller CS, et al Combination Pod-intravaginal Ring Delivers Antiretroviral Agents for HIV Prophylaxis: Pharmacokinetic Evaluation in an Ovine Model. Antimicrob Agents Chemother. 2016;60(6):3759–66. 10.1128/AAC.00391-16 27067321 PMC4879417

[30] Nugent RP, Krohn MA, Hillier SL. Reliability of Diagnosing Bacterial Vaginosis Is Improved by a Standardized Method of Gram Stain Interpretation. J Clin Microbiol. 1991;29(2):297–301. PubMed PMID: WOS:A1991ET57800014. 1706728 10.1128/jcm.29.2.297-301.1991 PMC269757

[31] Pyles RB, Vincent KL, Baum MM, Elsom B, Miller AL, Maxwell C, et al Cultivated Vaginal Microbiomes Alter HIV-1 Infection and Antiretroviral Efficacy in Colonized Epithelial Multilayer Cultures. PLoS ONE. 2014;9(3):e93419 PubMed Central PMCID: PMC3968159. 10.1371/journal.pone.0093419 24676219 PMC3968159

[32] Hendrix CW, Chen BA, Guddera V, Hoesley C, Justman J, Nakabiito C, et al MTN-001: Randomized Pharmacokinetic Cross-over Study Comparing Tenofovir Vaginal Gel and Oral Tablets in Vaginal Tissue and Other Compartments. PLoS ONE. 2013;8(1):e55013 10.1371/journal.pone.0055013 23383037 PMC3559346

[33] Parsons TL, Emory JF, Seserko LA, Aung WS, Marzinke MA. Dual Quantification of Dapivirine and Maraviroc in Cervicovaginal Secretions from Ophthalmic Tear Strips and Polyester-based Swabs via Liquid Chromatographic-tandem Mass Spectrometric (LC-MS/MS) Analysis. J Pharm Biomed Anal. 2014;98:407–16. 10.1016/j.jpba.2014.06.018 PubMed PMID: WOS:000339859800055. 25005891 PMC4143664

[34] Hendrix CW, Andrade A, Bumpus NN, Kashuba AD, Marzinke MA, Moore A, et al Dose Frequency Ranging Pharmacokinetic Study of Tenofovir-Emtricitabine After Directly Observed Dosing in Healthy Volunteers to Establish Adherence Benchmarks (HPTN 066). AIDS Res Hum Retrovir. 2016;32(1):32–43. 10.1089/AID.2015.0182 PubMed PMID: WOS:000367335100006. 26414912 PMC4692123

[35] Parsons TL, Marzinke MA. Development and Validation of a Liquid Chromatographic-tandem Mass Spectrometric Method for the Multiplexed Quantification of Etravirine, Maraviroc, Raltegravir, and Rilpivirine in Human Plasma and Tissue. J Pharm Biomed Anal. 2016;131:333–44. 10.1016/j.jpba.2016.08.016 PubMed PMID: WOS:000387628400044. 27632783

[36] US FDA. Guidance for Industry: Bioanalytical Method Validation Rockville, MD: U.S. Department of Health and Human Services, Food and Drug Administration, Center for Drug Evaluation and Research (CDER), Center for Veterinary Medicine (CVM); 2001 p. 22.

[37] Churchman SA, Moss JA, Baum MM. Accurate Measurement of Female Genital Tract Fluid Dilution in Cervicovaginal Lavage Samples. J Chromatogr B. 2016;1017:75–81. 10.1016/j.jchromb.2016.02.033 PMC4808354 26950030

[38] Guest G, MacQueen KM, Namey EE. Applied Thematic Analysis Thousand Oaks: SAGE Publications; 2012. 360 p.

[39] Guthrie KM, Rosen RK, Vargas SE, Getz ML, Dawson L, Guillen M, et al User Evaluations Offer Promise for Pod-intravaginal Ring as a Drug Delivery Platform: A Mixed Methods Study of Acceptability and Use Experiences. PLoS ONE. 2018;13(5):e0197269 10.1371/journal.pone.0197269 29758049 PMC5951541

[40] Moss JA, Baum MM, Malone AM, Kennedy S, Kopin E, Nguyen C, et al Tenofovir and Tenofovir Disoproxil Pharmacokinetics from Intravaginal Rings. AIDS. 2012;26(6):707–10. PubMed Central PMCID: PMC3855348. 10.1097/QAD.0b013e3283509abb 22210639 PMC3855348

[41] Belec L, Meillet D, Levy M, Georges A, Tevibenissan C, Pillot J. Dilution Assessment of Cervicovaginal Secretions Obtained by Vaginal Washing for Immunological Assays. Clin Diagn Lab Immunol. 1995;2(1):57–61. PubMed PMID: WOS:A1995QA64900011. 7719914 10.1128/cdli.2.1.57-61.1995 PMC170101

[42] Baeten JM, Donnell D, Ndase P, Mugo NR, Campbell JD, Wangisi J, et al Antiretroviral Prophylaxis for HIV Prevention in Heterosexual Men and Women. N Engl J Med. 2012;367(5):399–410. 10.1056/NEJMoa1108524 PubMed PMID: WOS:000307001900005. 22784037 PMC3770474

[43] Keller MJ, Mesquita PM, Marzinke MA, Teller R, Espinoza L, Atrio JM, et al A Phase 1 Randomized Placebo-controlled Safety and Pharmacokinetic Trial of a Tenofovir Disoproxil Fumarate Vaginal Ring. AIDS. 2016;30(5):743–51. 10.1097/QAD.0000000000000979 PubMed PMID: WOS:000371309700001. 26605514 PMC4767579

[44] Louissaint NA, Cao YJ, Skipper PL, Liberman RG, Tannenbaum SR, Nimmagadda S, et al Single Dose Pharmacokinetics of Oral Tenofovir in Plasma, Peripheral Blood Mononuclear Cells, Colonic Tissue, and Vaginal Tissue. AIDS Res Hum Retroviruses. 2013;29(11):1443–50. 10.1089/AID.2013.0044 PubMed PMID: WOS:000326037500505. 23600365 PMC3809387

[45] Srinivasan P, Moss JA, Gunawardana M, Churchman SA, Yang F, Dinh CT, et al Topical Delivery of Tenofovir Disoproxil Fumarate and Emtricitabine from Pod-intravaginal Rings Protect Macaques from Multiple SHIV Exposures. PLoS ONE. 2016;11(6):e0157061 PubMed Central PMCID: PMC4898685. 10.1371/journal.pone.0157061 27275923 PMC4898685

[46] Smith JM, Rastogi R, Teller RS, Srinivasan P, Mesquita PM, Nagaraja U, et al Intravaginal Ring Eluting Tenofovir Disoproxil Fumarate Completely Protects Macaques from Multiple Vaginal Simian-HIV Challenges. Proc Natl Acad Sci U S A. 2013;110(40):16145–50. 10.1073/pnas.1311355110 24043812 PMC3791780

[47] Dorr P, Westby M, Dobbs S, Griffin P, Irvine B, Macartney M, et al Maraviroc (UK-427,857), a Potent, Orally Bioavailable, and Selective Small-molecule Inhibitor of Chemokine Receptor CCR5 with Broad-spectrum Anti-human Immunodeficiency Virus Type 1 Activity. Antimicrob Agents Chemother. 2005;49(11):4721–32. 10.1128/AAC.49.11.4721-4732.2005 PubMed PMID: ISI:000233020900039. 16251317 PMC1280117

[48] Chen BA, Panther L, Marzinke MA, Hendrix CW, Hoesley CJ, van der Straten A, et al Phase 1 Safety, Pharmacokinetics, and Pharmacodynamics of Dapivirine and Maraviroc Vaginal Rings: A Double-blind Randomized Trial. J Acquir Immune Defic Syndr. 2015;70(3):242–9. 10.1097/QAI.0000000000000702 26034880 PMC4607587

[49] Baeten JM, Palanee-Phillips T, Brown ER, Schwartz K, Soto-Torres LE, Govender V, et al Use of a Vaginal Ring Containing Dapivirine for HIV-1 Prevention in Women. N Engl J Med. 2016;375(22):2121–32. 10.1056/NEJMoa1506110 ; PubMed Central PMCID: PMC4993693. 26900902 PMC4993693

[50] Nel A, van Niekerk N, Kapiga S, Bekker LG, Gama C, Gill K, et al Safety and Efficacy of a Dapivirine Vaginal Ring for HIV Prevention in Women. N Engl J Med. 2016;375(22):2133–43. 10.1056/NEJMoa1602046 . 27959766

[51] Brady M, Manning J. Lessons from Reproductive Health to Inform Multipurpose Prevention Technologies: Don't Reinvent the Wheel. Antiviral Res. 2013;100:S25–S31. 10.1016/j.antiviral.2013.09.019 PubMed PMID: WOS:000328923200005. 24188700

[52] Boonstra H, Barot S, Lusti-Narasimhan M. Making the Case for Multipurpose Prevention Technologies: the Socio-epidemiological Rationale. BJOG. 2014;121:23–6. 10.1111/1471-0528.12851 PubMed PMID: WOS:000344399300007. 25335837

[53] Woodsong C, Holt JDS. Acceptability and Preferences for Vaginal Dosage Forms Intended for Prevention of HIV or HIV and Pregnancy. Adv Drug Deliv Rev. 2015;92:146–54. 10.1016/j.addr.2015.02.004 PubMed PMID: WOS:000364269500011. 25703190

[54] Friend DR. An Update on Multipurpose Prevention Technologies for the Prevention of HIV Transmission and Pregnancy. Expert Opin Drug Deliv. 2016;13(4):533–45. PubMed PMID: WOS:000372075500004. 10.1517/17425247.2016.1134485 26742698

[55] Quaife M, Terris-Prestholt F, Vickerman P. The Promise of Multipurpose Pregnancy, STI, and HIV Prevention. Lancet Infect Dis. 2017;17(1):21–2. PubMed PMID: WOS:000389864700021. 10.1016/S1473-3099(16)30550-3 27998564

[56] Baeten J, Heffron R, Kidoguchi L, Mugo N, Katabira E, Bukusi E, et al., editors. Near Elimination of HIV Transmission in a Demonstration Project of PrEP and ART. 2015 Conference on Retroviruses and Opportunistic Infections (CROI); 2015 Feb. 23–26, 2015; Seattle, WA: CROI, Alexandria, VA.

[57] Amico KR, Wallace M, Bekker LG, Roux S, Atujuna M, Sebastian E, et al Experiences with HPTN 067/ADAPT Study Provided Open-Label PrEP Among Women in Cape Town: Facilitators and Barriers Within a Mutuality Framework. AIDS Behav. 2017;5:1361–75. 10.1007/s10461-016-1458-y PMC5378745 27317411

[58] Molina J-M, Charreau I, Spire B, Cotte L, Pialoux, Capitant C, et al., editors. On Demand PrEP With Oral TDF-FTC in the Open-Label Phase of the ANRS IPERGAY Trial. 2016 Conference on Retroviruses and Opportunistic Infections (CROI); 2016 Feb. 22–25, 2016; Boston, MA: CROI, Alexandria, VA.

