# Supplementary figures and images for: Safety and pharmacokinetics of single, dual, and triple antiretroviral drug formulations delivered by pod-intravaginal rings designed for HIV-1 prevention: A Phase I trial
Source: PLoS Med. 2018 Sep 28;15(9):e1002655. doi: 10.1371/journal.pmed.1002655 (PMC6161852; doi:10.1371/journal.pmed.1002655)

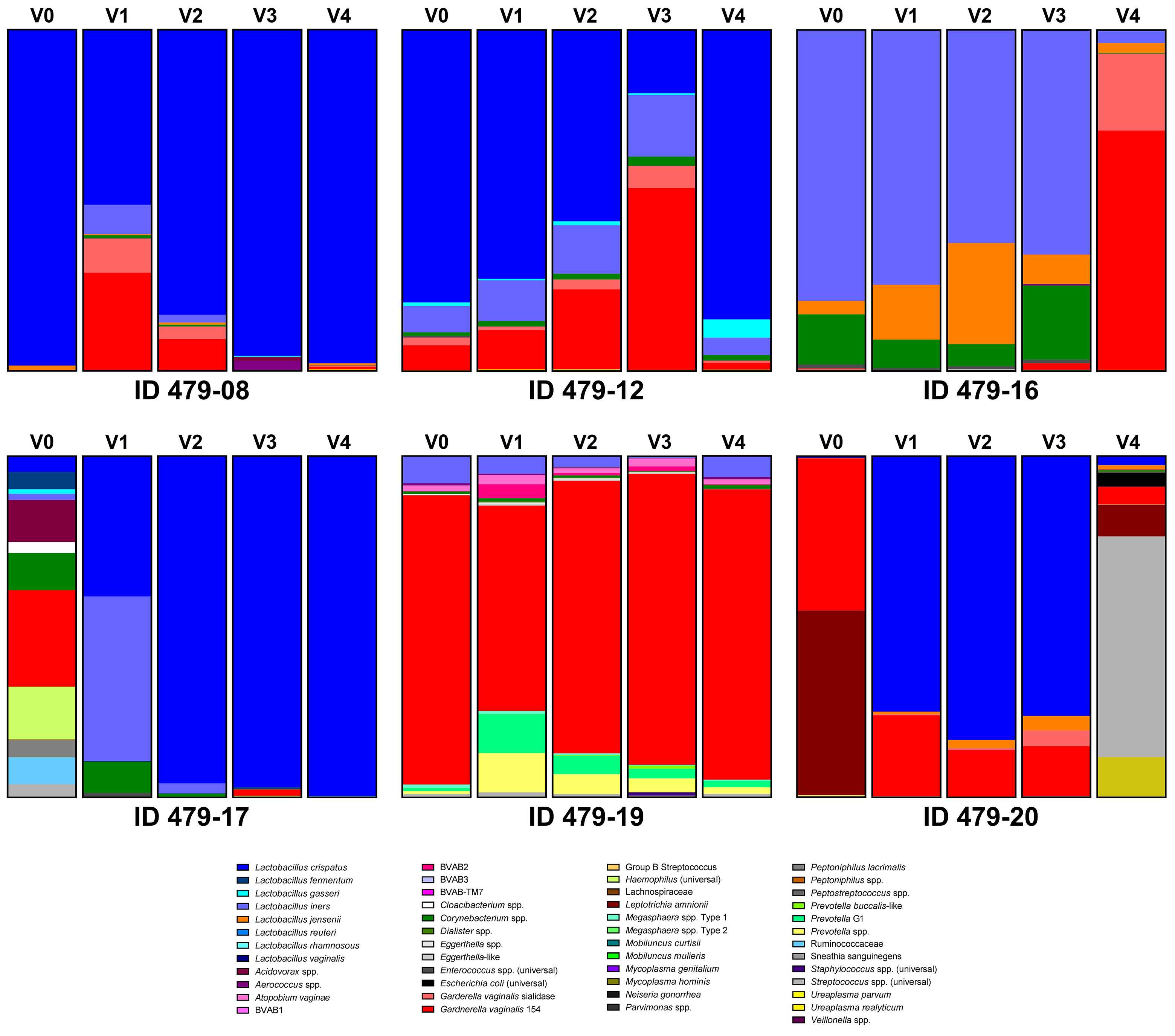

Supplement: S1 Fig — The IVRs were inserted at V1 and removed at V3. IVR, intravaginal ring; qPCR, quantitative polymerase chain reaction; TDF, tenofovir disoproxil fumarate. (TIF) [file pmed.1002655.s002.tif]

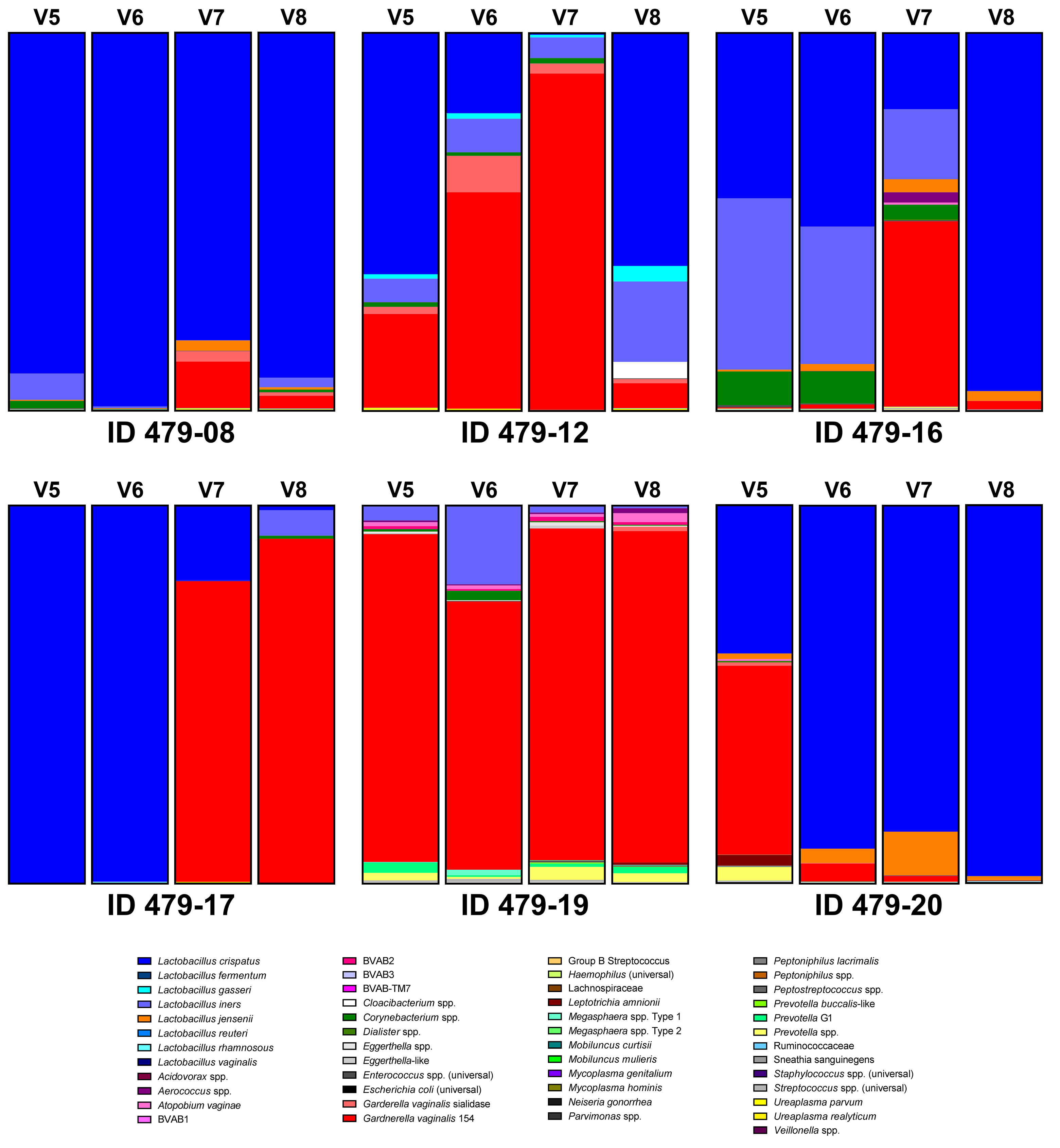

Supplement: S2 Fig — The IVRs were inserted at V5 and removed at V7. FTC, emtricitabine; IVR, intravaginal ring; qPCR, quantitative polymerase chain reaction; TDF, tenofovir disoproxil fumarate. (TIF) [file pmed.1002655.s003.tif]

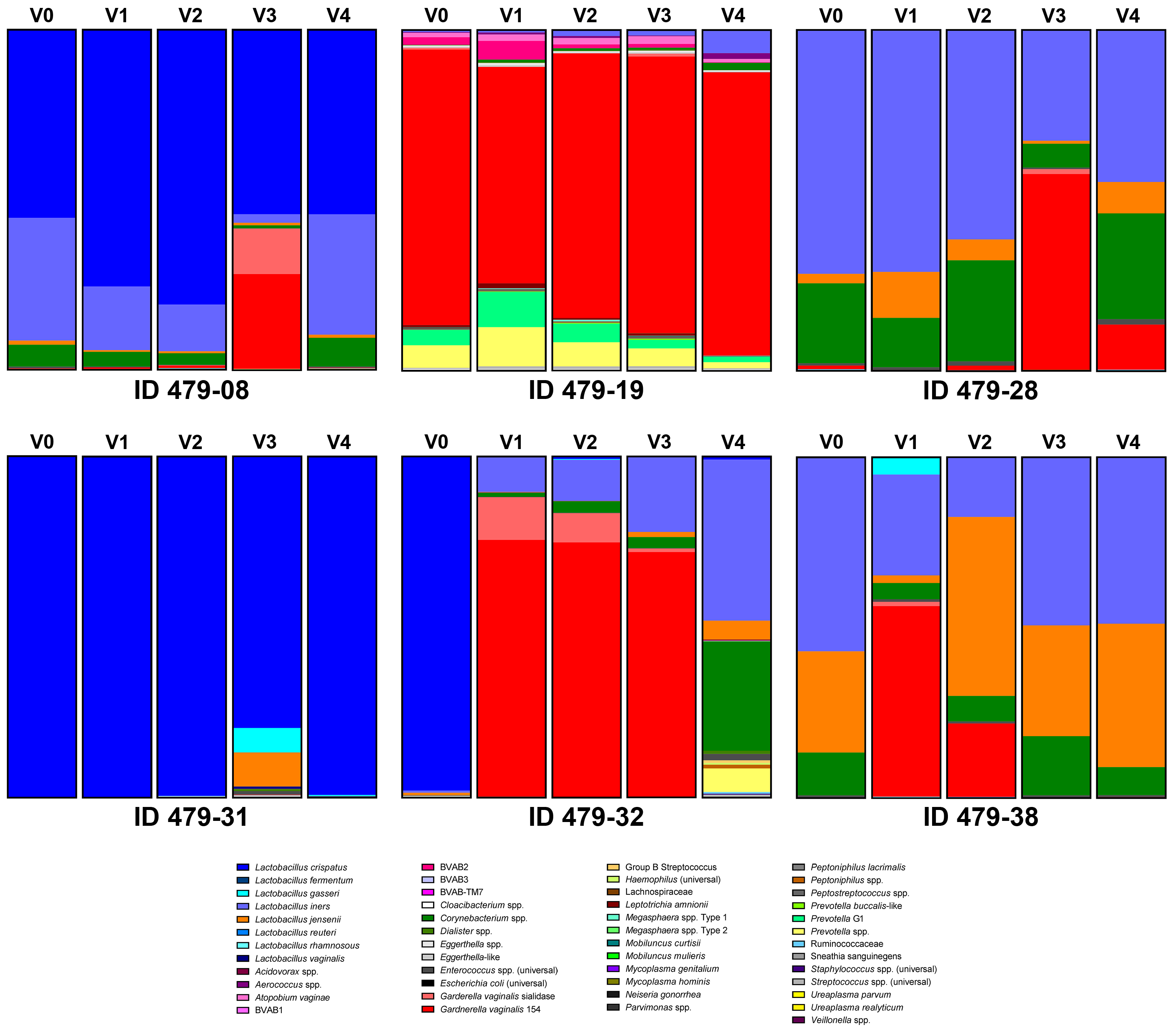

Supplement: S3 Fig — The IVRs were inserted at V1 and removed at V3. FTC, emtricitabine; IVR, intravaginal ring; MVC, maraviroc; qPCR, quantitative polymerase chain reaction; TDF, tenofovir disoproxil fumarate. (TIF) [file pmed.1002655.s004.tif]

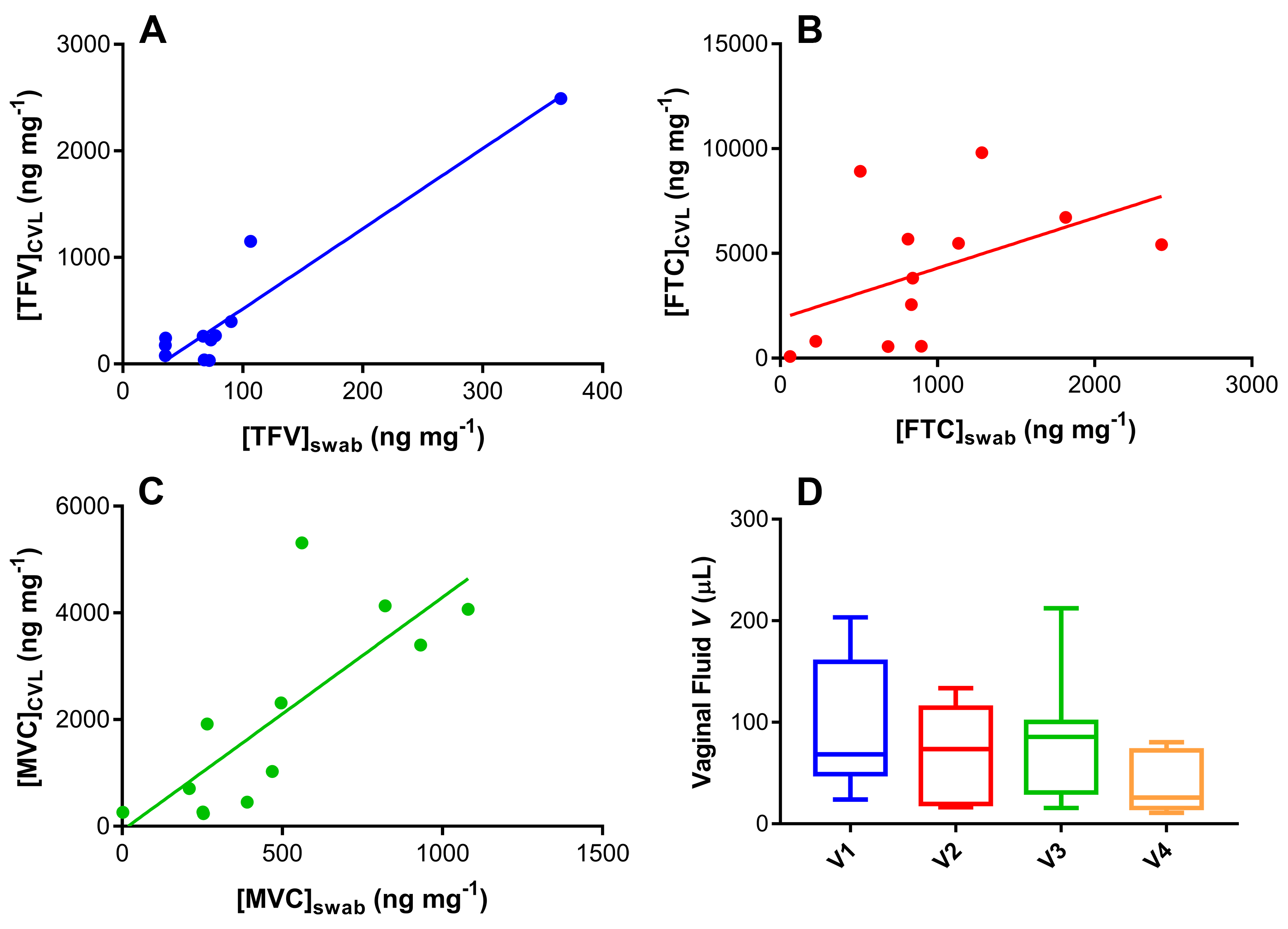

Supplement: S4 Fig — Drug concentrations with the IVR in place (V2–V3) in CVL samples corrected for CVF dilution (y-axis) plotted against paired drug concentrations in neat CVF samples collected using Dacron swabs (x-axis) exhibit a moderate-weak correlation, with systematically higher values in CVL samples. (A) TFV (total TFV, reported as the molar sum of TDF and TFV concentrations in the samples); slope, 7.53 ± 0.84; R2, 0.889; FTC; slope, 2.40 ± 1.43; R2, 0.220; MVC; slope, 4.37 ± 1.11; R2, 0.609. (D) Box plots of CVF volume collected for all participants (n = 6) at each study visit (V1–V4). The box extends from the 25th to 75th percentiles, with the horizontal line in the box representing the median; whiskers represent the lowest and highest datum. CVF, cervicovaginal fluid; CVL, cervicovaginal lavage; FTC, emtricitabine; IVR, intravaginal ring; MVC, maraviroc; TDF, tenofovir disoproxil fumarate; TFV, tenofovir. (TIF) [file pmed.1002655.s005.tif]
