# Supplementary material for: Safety and pharmacokinetics of single, dual, and triple antiretroviral drug formulations delivered by pod-intravaginal rings designed for HIV-1 prevention: A Phase I trial
Source: PLoS Med. 2018 Sep 28;15(9):e1002655. doi: 10.1371/journal.pmed.1002655 (PMC6161852; doi:10.1371/journal.pmed.1002655)
Supplement: S1 Table — IVR, intravaginal ring; TDF, tenofovir disoproxil fumarate. (DOCX) [file pmed.1002655.s006.docx]

**S1 Table. Summary of drug and drug metabolite concentrations in key anatomic compartments measured with TDF pod-IVR in place (six participants); i.e., Visits 2 and 3.**

Measurements outside of the analytical ranges were not included in the analysis.

| **IVR, analyte, matrix** | ***n^a^*** | **% > LLQ^b^** | **Median (IQR)^c^** |
| --- | --- | --- | --- |
| TDF, CVF ^d^, ng mg^-1^ | 12 | 92 | 58.1 (43.9-97.4) |
| TFV, CVF, ng mg^-1^ | 12 | 83 | 13.9 (6.2-19.3) |
| TDF, CVL^e^ ng mL^-1^ | 13 | 100 | 1,930 (1,040-2,290) |
| TFV, CVL, ng mL^-1^ | 13 | 100 | 611 (242-2,440) |
| TFV, VT ^f^, ng mg^-1^ | 6 | 100 | 8.4 (4.7-11.2) |
| TFV-DP, VT, fmol mg^-1^ | 6 | 83 | 303 (277-938) |
| TFV, plasma, ng mL^-1^ | 13 | 0 | N/A^g^ |

^a^ Number of samples analyzed.

^b^ LLQ=lower limit of quantification; Data represent proportions of samples that contained quantifiable drug levels.

^c^ IQR= Interquartile range (25th to 75th percentile).

^d^ CVF= cervicovaginal fluid

^e^ CVL= cervicovaginal lavage; Measurements not compensated for dilution during the CVL procedure.

^f^ VT=vaginal tissue

^g^ Not applicable.
