# Supplementary material for: Safety and pharmacokinetics of single, dual, and triple antiretroviral drug formulations delivered by pod-intravaginal rings designed for HIV-1 prevention: A Phase I trial
Source: PLoS Med. 2018 Sep 28;15(9):e1002655. doi: 10.1371/journal.pmed.1002655 (PMC6161852; doi:10.1371/journal.pmed.1002655)
Supplement: S2 Table — FTC, emtricitabine; IVR, intravaginal ring; TDF, tenofovir disoproxil fumarate. (DOCX) [file pmed.1002655.s007.docx]

**S2 Table. Summary of drug and drug metabolite concentrations in key anatomic compartments measured with TDF-FTC pod-IVR in place (six participants); i.e., Visits 6 and 7.**

Measurements outside of the analytical ranges were not included in the analysis.

| **IVR, analyte, matrix** | ***n^a^*** | **% > LLQ^b^** | **Median (IQR)^c^** |
| --- | --- | --- | --- |
| TDF, CVF **^d^**, ng mg^-1^ | 12 | 100 | 43.1 (31.0-65.0) |
| TFV, CVF, ng mg^-1^ | 12 | 100 | 15.9 (7.1-20.0) |
| FTC, CVF, ng mg^-1^ | 12 | 100 | 896 (367-1349) |
| TDF, CVL**^e^**, ng mL^-1^ | 12 | 100 | 1,720 (1,028-3,429) |
| TFV, CVL, ng mL^-1^ | 12 | 100 | 927 (315-1,253) |
| FTC, CVL, ng mL^-1^ | 12 | 100 | 23,950 (11,373-56,400) |
| TFV, VT **^f^**, ng mg^-1^ | 6 | 100 | 5.1 (0.8-10.1) |
| TFV-DP, VT, fmol mg^-1^ | 6 | 67 | 289 (110-603) |
| FTC, VT, ng mg^-1^ | 6 | 100 | 74.5 (11.6-193) |
| TFV, plasma, ng mL^-1^ | 12 | 0 | N/A^g^ |
| FTC, plasma, ng mL^-1^ | 12 | 50 | 0.95 (0.91-1.14) |

^a^ Number of samples analyzed.

^b^ LLQ=lower limit of quantification; Data represent proportions of samples that contained quantifiable drug levels.

^c^ IQR= Interquartile range (25th to 75th percentile).

^d^ CVF= cervicovaginal fluid

^e^ CVL= cervicovaginal lavage; Measurements not compensated for dilution during the CVL procedure.

^f^ VT=vaginal tissue

^g^ Not applicable.
