# Supplementary material for: Safety and pharmacokinetics of single, dual, and triple antiretroviral drug formulations delivered by pod-intravaginal rings designed for HIV-1 prevention: A Phase I trial
Source: PLoS Med. 2018 Sep 28;15(9):e1002655. doi: 10.1371/journal.pmed.1002655 (PMC6161852; doi:10.1371/journal.pmed.1002655)
Supplement: S3 Table — FTC, emtricitabine; IVR, intravaginal ring; MVC, maraviroc; TDF, tenofovir disoproxil fumarate. (DOCX) [file pmed.1002655.s008.docx]

**S3 Table. Summary of drug and drug metabolite concentrations in key anatomic compartments measured with TDF-FTC-MVC pod-IVR in place (six participants); i.e., Visits 2 and 3.**

Measurements outside of the analytical ranges were not included in the analysis.

| **IVR, analyte, matrix** | ***n^a^*** | **% > LLQ^b^** | **Median (IQR)^c^** |
| --- | --- | --- | --- |
| TDF, CVF **^d^**, ng mg^-1^ | 12 | 100 | 96.9 (14.5-137.1) |
| TFV, CVF, ng mg^-1^ | 12 | 100 | 28.0 (24.3-31.9) |
| FTC, CVF, ng mg^-1^ | 12 | 100 | 838.5 (641.8-1171.2) |
| MVC, CVF, ng mg^-1^ | 12 | 100 | 429.8 (253.2-626.2) |
| TDF, CVL **^e^**, ng mL^-1^ | 12 | 92 | 3630 (2310-15700) |
| TFV, CVL, ng mL^-1^ | 12 | 100 | 2670 (1668-4175) |
| FTC, CVL, ng mL^-1^ | 12 | 100 | 72700 (43425-197500) |
| MVC, CVL, ng mL^-1^ | 12 | 100 | 32850 (16000-88125) |
| TFV, VT **^f^**, ng mg^-1^ | 6 | 100 | 5.1 (3.3-9.7) |
| TFV-DP, VT, fmol mg^-1^ | 6 | 100 | 301.9 (177.1-823.8) |
| FTC, VT, ng mg^-1^ | 6 | 100 | 104.0 (63.7-301.7) |
| MVC, VT, ng mg^-1^ | 6 | 100 | 141.8 (82.5-212.0) |
| TFV, plasma, ng mL^-1^ | 12 | 0 | N/A ^g^ |
| FTC, plasma, ng mL^-1^ | 12 | 83 | 1.02 (0.56-1.22) |
| MVC, plasma, ng mL^-1^ | 12 | 50 | 0.08 (0.05-0.14) |

^a^Number of samples analyzed.

^b^ LLQ=lower limit of quantification; Data represent proportions of samples that contained quantifiable drug levels.

^c^ IQR= Interquartile range (25th to 75th percentile).

^d^ CVF= cervicovaginal fluid

^e^ CVL= cervicovaginal lavage; Measurements not compensated for dilution during the CVL procedure.

^f^ VT=vaginal tissue

^g^ Not applicable.
